# Resistance to BRAF inhibition explored through single circulating tumour cell molecular profiling in BRAF-mutant non-small-cell lung cancer

**DOI:** 10.1038/s41416-023-02535-0

**Published:** 2024-01-04

**Authors:** Laura Mezquita, Marianne Oulhen, Agathe Aberlenc, Marc Deloger, Mihaela Aldea, Aurélie Honore, Yann Lecluse, Karen Howarth, Luc Friboulet, Benjamin Besse, David Planchard, Françoise Farace

**Affiliations:** 1 Gustave Roussy, Université Paris-Saclay, Department of Medicine, F-94805 Villejuif, France; 2 Medical Oncology Department, Hospital Clinic of Barcelona, Laboratory of Translational Genomics and Targeted Therapies in Solid Tumors, IDIBAPS, Barcelona, Spain; 3 Gustave Roussy, Université Paris-Saclay, “Rare Circulating Cells” Translational Platform, CNRS UMS3655—INSERM US23 AMMICA, F-94805 Villejuif, France; 4 INSERM, U981 “Identification of Molecular Predictors and new Targets for Cancer Treatment”, F-94805 Villejuif, France; 5 Gustave Roussy, Université Paris-Saclay, Bioinformatics Platform, CNRS UMS3655—INSERM US23 AMMICA, F-94805 Villejuif, France; 6 Gustave Roussy, Université Paris-Saclay, Genomic Platform, CNRS UMS3655—INSERM US23 AMMICA, F-94805 Villejuif, France; 7 Gustave Roussy, Université Paris-Saclay, “Flow cytometry and Imaging” Platform, CNRS UMS3655—INSERM US23AMMICA, F-94805 Villejuif, France; 8 Inivata Ltd, Babraham Research Park, Cambridge, UK

**Keywords:** Molecular medicine, Non-small-cell lung cancer

## Abstract

**Background:**

Resistance mechanisms to combination therapy with dabrafenib plus trametinib remain poorly understood in patients with *BRAF*^V600E^-mutant advanced non-small-cell lung cancer (NSCLC). We examined resistance to BRAF inhibition by single CTC sequencing in *BRAF*^V600E^-mutant NSCLC.

**Methods:**

CTCs and cfDNA were examined in seven *BRAF*^V600E^-mutant NSCLC patients at failure to treatment. Matched tumour tissue was available for four patients. Single CTCs were isolated by fluorescence-activated cell sorting following enrichment and immunofluorescence (Hoechst 33342/CD45/pan-cytokeratins) and sequenced for mutation and copy number-alteration (CNA) analyses.

**Results:**

*BRAF*^V600E^ was found in 4/4 tumour biopsies and 5/7 cfDNA samples. CTC mutations were mostly found in MAPK-independent pathways and only 1/26 CTCs were *BRAF*^V600E^ mutated. CTC profiles encompassed the majority of matched tumour biopsy CNAs but 72.5% to 84.5% of CTC CNAs were exclusive to CTCs. Extensive diversity, involving MAPK, MAPK-related, cell cycle, DNA repair and immune response pathways, was observed in CTCs and missed by analyses on tumour biopsies and cfDNA. Driver alterations in clinically relevant genes were recurrent in CTCs.

**Conclusions:**

Resistance was not driven by *BRAF*^V600E^-mutant CTCs. Extensive tumour genomic heterogeneity was found in CTCs compared to tumour biopsies and cfDNA at failure to BRAF inhibition, in *BRAF*^V600E^-mutant NSCLC, including relevant alterations that may represent potential treatment opportunities.

## Introduction

Lung cancer is the most common cause of cancer-related death worldwide, owing to its metastatic spread at the time of diagnosis [1]. The molecular characterisation of Non-Small-Cell Lung Cancer (NSCLC) and discovery of oncogene driver alterations have revolutionised the therapeutic landscape of NSCLC. Molecularly targeted therapy using tyrosine kinase inhibitors (TKIs) has led to major clinical improvement in about 25% of patients with NSCLC harbouring *epidermal growth factor receptor* (*EGFR*) activating alterations, *anaplastic lymphoma kinase* (*ALK*) gene or *c-ros oncogene 1 (ROS1*) fusions [2]. More recently, *BRAF* mutations—responsible for the constitutive activation of mitogen-activated protein kinase (MAPK)/extracellular signal-regulated kinase (ERK) pathway—have emerged as a novel molecular target in around 2% of NSCLC patients [3, 4]. Initial studies demonstrated the clinical activity of selective inhibitors dabrafenib or vemurafenib as single agents in previously treated patients with *BRAF*^V600E^-mutant NSCLC, observed in 50% of patients with a *BRAF* mutation [5, 6]. Similar to melanoma, superior efficacy of combined BRAF and MEK inhibition compared to BRAF inhibitor monotherapy was observed in *BRAF*^V600E^-mutant NSCLC. The combination of dabrafenib and MEK inhibitor trametinib produced substantial antitumor activity (ORR, 66.7%) with durable responses (median PFS, 10.2 months) in previously treated *BRAF*^V600E^*-*mutant NSCLC patients [5]. Moreover, significant clinical improvement of this combination therapy over both single-agent dabrafenib and conventional chemotherapy was observed in untreated *BRAF*^V600E^ NSCLC [6]. These studies have led to the European Medicines Agency and US Food and Drug Administration approval of dabrafenib-trametinib combination for the treatment of *BRAF*^V600E^-mutant NSCLC and its recent recommendation as upfront and standard of care treatment in this malignancy.

Nevertheless, in spite of high objective response rates, acquired resistance to targeted therapy inevitably develops, leading to disease progression in patients with *BRAF*^V600E^-mutant NSCLC. Knowledge about resistance mechanisms to BRAF inhibition results mainly from studies conducted in metastatic melanoma. Very limited data are available for NSCLC so far. Unlike *EGFR* or *ALK*, acquired resistance mutations within the *BRAF* gene remain to be elucidated. In melanoma, it has been proposed that the development of secondary resistance mechanisms can be due to (1) ERK reactivation through the MAPK pathway, (2) bypass signalling tracks leading to constitutive activation of alternative oncogenic pathways, (3) other unknown mechanisms [7–9]. Reactivation of ERK upstream or downstream of BRAF kinase constitutes the main secondary resistance mechanism to BRAF inhibition in metastatic melanoma. Activation of bypass pathways such as PI3K-AKT represents another critical mechanism of acquired resistance in melanoma. In NSCLC, mechanisms of ERK reactivation mainly involved *BRAF* variants, *BRAF* gene amplification or secondary mutations in other genes of the MAPK/ERK signalling pathway such as *NRAS/KRAS* or *MEK* mutations, leading to BRAF-independent reactivation of ERK signalling [10]. Mechanisms of secondary resistance to dual inhibition of BRAF and MEK are more complex but in most cases also involve the reactivation of MAPK pathway and ERK signalling as observed for single-agent resistance [11–14].

Genomic studies of primary tumours and metastases have unravelled the complex and heterogeneous molecular landscape of NSCLC and its implication in response to therapy. Liquid biopsy components such as circulating tumour cells (CTCs) and cell-free (cf) DNA are likely released from primary tumour or spatially distinct metastatic sites and provide a minimally invasive tool to investigate tumour genomic content. CfDNA has emerged as an effective tool for monitoring genetic alterations predictive of tumour relapse and tracking resistance mutations in NSCLC patients progressing under targeted therapies [15, 16]. In contrast to cfDNA, CTCs may contain the most aggressive cell clones highly relevant in metastatic progression. CTC sequencing at the single-cell level provides the opportunity to identify distinct tumour cell clones, assess actionable alterations and emerging resistant subclones and potentially uncover the role of tumour heterogeneity in therapeutic failure and drug resistance. Despite the rare nature of CTCs, technological advances have fuelled translational research studies leading to demonstrate their clinical utility and identify predictive biomarkers of therapeutic sensitivity and resistance [17–22]. Recently, the feasibility of genomic profiling of single CTCs has been reported in several tumour types, including lung cancer [23–25]. In *ALK*-rearranged NSCLC, we showed that sequencing single CTCs unravelled both “on-target and off-target” acquired genomic alterations to ALK-TKIs, providing new insight into the therapeutic resistance landscape in this patient subset [26, 27].

Here, in a pilot study, we performed molecular profiling of single CTCs and cfDNA from seven patients with *BRAF*^V600E^-mutant NSCLC to identify genetic alterations (mutations, copy number alterations (CNAs)) occurring at disease progression to combined dabrafenib-trametinib treatment. These profiles inform on therapeutic resistance in *BRAF-*mutant patients and provide a proof-of-concept of the clinical utility of liquid biopsies in this setting.

## Materials and methods

### Patients

Seven patients with advanced *BRAF*^V600E^-mutant NSCLC were prospectively enrolled and monitored on dabrafenib-trametinib treatment between May 2018 and November 2019 at Gustave Roussy (Villejuif, France). The MATCH-R study (NCT02517892) was conducted in accordance with the Declaration of Helsinki. It was authorised by the French national regulation agency ANSM (*Agence Nationale de Sécurité du Medicament et des produits de santé*) and approved by the Ethics Committee and our institutional review board. Informed written consent was obtained from all patients. *BRAF*^V600E^ mutation was detected in diagnostic tumour specimens undergoing routine NSCLC molecular testing (*EGFR, ALK, ROS1, BRAF, KRAS, HER2, PI3K, MET*). Clinical, pathological and molecular data were collected from the electronic medical records. Tumour tissue was obtained for five patients at radiological progressive disease (PD). Blood samples (40 mL) were collected in CellSave tubes (Menarini Silicon Biosystems, #CS0018) for CTC analysis, and 10 mL of blood was collected in a Streck tube (Streck, #218962) for cfDNA. Because the treatment could potentially have an impact on CTC numbers, blood sampling for CTC analyses was performed at the end of treatment.

### CfDNA isolation and analysis

CfDNA isolation and analysis is described in the Supplementary Methods section.

### Tissue DNA sequencing

Tissue DNA sequencing is described in the Supplementary Methods section.

### Statistical analysis

Overall survival (OS) was calculated from the date of systemic therapy initiation until death due to any cause. Progression-free survival (PFS) was calculated from the date of each systemic therapy initiation until progression (PD) or death due to any cause. PD was assessed as per RECIST v1.1, except for one patient (P3, non-measurable bone disease). Statistical analysis was performed using R.

### CTC enumeration by the CellSearch

CTCs were enumerated using CellSearch (Menarini Silicon Biosystems, Bologna, Italy) as previously reported [28].

### CTC enrichment, immunofluorescence staining and isolation

Negative selection of CTCs was performed using the RosetteSep Human CD36 Depletion Cocktail (StemCell Technologies, #15167) according to the manufacturer’s protocol. After washing, the CTC-enriched cell fraction was fixed and permeabilized using the Fix&Perm kit (Thermo Fisher Scientific, #GAS004). During permeabilization, cells were stained with 50 µL of cytokeratins-PE (cytokeratins 8, 18, 19) and CD45-APC antibodies from the CellSearch reagent kit (Menarini Silicon Biosystem, #CS0009) for 20 min in the dark at room temperature. After a PBS 1× wash, the cell pellet was re-suspended in 300 µL of PBS 1× and kept at +4 °C. Hoechst 33342 (Sigma Aldrich, #14533-100MG) was added before cell sorting. Individual CTC isolation was performed using a BD FACS ARIA III cell sorter (BD Biosciences) equipped with four lasers (a 405-nm laser, a 488-nm laser, a 561-nm laser and a 640-nm laser). The system was run with 20 psi pressure, a 100-µm nozzle and the yield precision mode. The first gate included Hoechst-positive elements. The second gate enabled selecting CD45-APC-negative events. Individual Hoechst^+^/CD45-APC^-^/CK-PE^+^ cells were sorted and collected in a 96-well plate. As a control, 50 Hoechst^+^/CD45-APC^+^/CK-PE^−^ cells were sorted in a well. Plates were centrifuged 10 min at 1200 rpm and frozen at −20 °C for at least 30 min.

### Cell line

Colon cancer COLO 205 cell line was obtained from the ATCC and cultured in standard conditions.

### Whole-genome amplification (WGA), quality controls (QC) and double-stranded (ds)DNA conversion

WGA was performed using the Ampli1 WGA kit (Menarini Silicon Biosystems) according to the manufacturer’s instructions. The quality of Ampli1 WGA products was checked as previously reported [29]. To increase the total dsDNA content in Ampli1 WGA products, single-strand (ss)DNA molecules were converted into dsDNA molecules using the Ampli1 ReAmp/ds kit (Menarini Silicon Biosystems).

### Isolation of genomic DNA from blood and tumour biopsies

Isolation of DNA from formalin-fixed paraffin-embedded tumour biopsies and whole blood is described in the Supplementary Methods section.

### Library preparation and ion torrent-targeted next-generation sequencing (NGS)

Ampli1 WGA products were cleaned up with 1.8X SPRIselect Beads (Beckman Coulter) and then quantified using Qubit fluorometer (Life Technologies) according to the manufacturer’s instructions. To analyse cancer gene sequence variants, the Ampli1 Cancer Hotspot Panel Custom Beta adapted from Ion Ampliseq CHP v2 by Menarini Silicon Biosystems covering 2265 COSMIC hotspot regions across 315 amplicons of 48 cancer-related genes commonly mutated in cancer was used as previously described [26].

### Library preparation and low-pass whole-genome sequencing (LP-WGS)

This workflow was done by Menarini Silicon Biosystems. Ampli1 LowPass kit for Illumina (Menarini Silicon Biosystems) was used for preparing LP-WGS libraries from single cells. For high-throughput processing, the manufacturer’s procedure was implemented in a fully automated workflow on a STARlet Liquid Handling Robot (Hamilton). Ampli1 LowPass libraries were normalised and sequenced by HiSeq 2500 instrument using 150 SR rapid-run mode.

### Bioinformatic workflow for targeted NGS

Sequence alignment and variant calling are described in the Supplementary Methods section.

### Bioinformatic workflow for LP-WGS

Sequence alignment, CNA calling, ploidy determination and hierarchical clustering are described in the Supplementary Methods section.

## Results

### Clinical characteristics

**Fig. 1:**
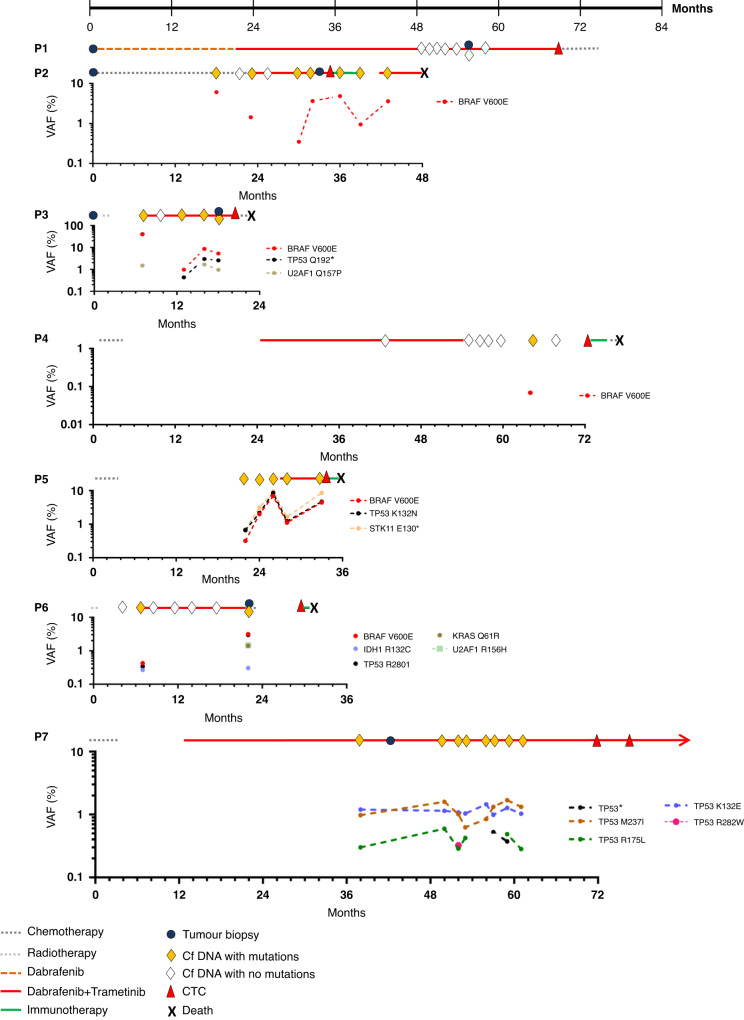
Timelines of treatments, tumour biopsies and blood sample collection for cfDNA and CTC analysis. CtDNA monitoring data is also provided. Only cfDNA mutations with a VAF threshold ≥ 0.25% are presented, except for *BRAF*^V600E^ mutation from patient P4 (VAF, 0.06875%).

Blood samples from seven NSCLC patients with *BRAF*^V600E^-mutant tumours were collected on combined dabrafenib and trametinib treatment. The main baseline characteristics of the study population are summarised in Table 1. The median age of the patient cohort was 68 years [range, 58–81 years]. All patients had adenocarcinoma and four were current or former smokers. Two patients (P3 and P6) received dabrafenib-trametinib as first-line treatment. The other five patients previously received chemotherapy or dabrafenib as monotherapy before the dabrafenib-trametinib combination. Overall, median OS in response to dabrafenib-trametinib was 37.4 months [95% confidence interval (CI); range, 11.5–72.4]. The median PFS was 16.1 months [95% CI; range, 6.9–44.8] and the median treatment duration was 16.65 months [range, 7.5–46.8], but all patients continued therapy beyond progression. One patient (P7) has been under dabrafenib-trametinib treatment for over eight years. Tumour biopsies were collected in five patients (P1, P2, P3, P6 and P7) at radiological progressive disease (PD). For patient P7, tumour biopsy harboured an insufficient tumour cell proportion and was thereby excluded from molecular analysis. CfDNA longitudinal monitoring was performed on treatment or within one week of treatment discontinuation and at PD. CTCs were analysed at PD only. For patients P1, P2, P3 and P6, the delay between tumour biopsy collection and blood sampling for CTC analyses was 12.8, 1.4, 2.8 and 9.1 months, respectively. Regarding P7, two blood samples were collected with a delay of 4.9 months and analysed (Table 1). The timelines of treatments, tumour and blood sample collection for cfDNA and CTC analysis and ctDNA monitoring data are presented in Fig. 1.

**Table 1:** Clinicobiological characteristics of *BRAF*^V600E^-mutated NSCLC patients.

Patient	Age at baseline (y/o)	Gender	Smoking status	Line of therapy	Best response	Number of metastatic sites at baseline	PD sites	Duration of treatment (months)	Progression-free survival (months)	Overall survival (months)	ECOG PS at time of CTC Isolation	Time between CTC - therapy initiation (months)	Detected CTCs by CellSearch at PD (/7.5 mL)	Isolated CTCs by FACS at PD (/30 mL)	Biopsy at PD
P1	65	F	Non-smoker	2nd	PR	≤2	Lung	46.8	44.8	72.4	1	46.8	0	25	Yes
P2	69	F	Smoker	2nd	PR	≤2	Adrenal	16.9	14.2	37.4	1	16.9	14	16	Yes
P3	58	M	Smoker	1st	SD	≤2	Bone peritoneal nodal	13.3	10.3	11.5	2	13.3	NA	23	Yes
P4	62	M	Smoker	2nd	PR	≤2	Bone	30.2	27.0	58.3	1	49.6	0*	17*	No
P5	68	M	Smoker	2nd	PR	>2	Liver brain	7.5	6.9	26.3	2	7.6	0	8	No
P6	81	F	Non-smoker	1st	SD	≤2	Liver bone	16.4	16.1	18.5	2	24.3	3*	28*	Yes
P7	69	M	Non-smoker	2nd	PR	≤2	Liver (oligoPD)	Ongoing	31.9	73.7	1 (0)**	60.9 (65.8)**	0 (0)**	22 (11)**	Yes***

*ECOG* Eastern Cooperative Oncology Group, *PS* performance status, *y/o* years old, *PD* progression disease.

*Isolated CTCs during a therapeutic break after BRAF/MEK inhibitor treatment.

**For patient P7, a second blood sample was collected 4.9 months after the first sample. Data for the second time point are presented in parentheses.

***Insufficient tumour cell proportion for molecular analysis.

### Mutational analysis of single CTCs, tumour biopsies and cfDNA

According to CellSearch, we detected a total of 17 CTCs (median, 0; [range, 0–14] at resistance to dabrafenib-trametinib (Table 1). In parallel, given the low sensitivity of CellSearch in NSCLC [17, 18, 30], we used hematopoietic blood-cell depletion combined to immunofluorescence staining and fluorescence-activated cell sorting (FACS) to isolate single Hoechst^+^/CD45^−^/Cytokeratins^+^ (called CD45^−^/CK^+^) cells from 30 mL blood samples, according to a previously reported experimental workflow [26, 27]. A total of 150 (median, 9.5; [range, 8–28]) single-candidate CTCs (CD45^−^/CK^+^ cells) were isolated according to this second strategy from the seven patients (Table 1). The majority of single-cell samples were subjected to WGA and quality control. 41/144 (28.5%) of tested samples showed a high Genome Integrity Index (GII) of 3 or 4 (Supplementary Fig. 1). We further included 19 samples with a GII of 2 to increase the data points on selected patients. A total of 60/144 (41.7%) single-cell samples were engaged in targeted NGS using a panel covering COSMIC hotspot regions of 48 cancer-related genes commonly mutated in cancer, as previously reported [26]. The whole molecular single-cell process was validated by testing the *BRAF*^V600E^-mutant cell line COLO 205 (Supplementary Table 1). Mean depth of sequencing over samples was 1823×. The median of amplicons with a depth ≥50× and the median of coverage uniformity were 64.5% [range, 9–87%] and 48.5% [range, 8–78%], respectively (Supplementary Table 2).

**Fig. 2:**
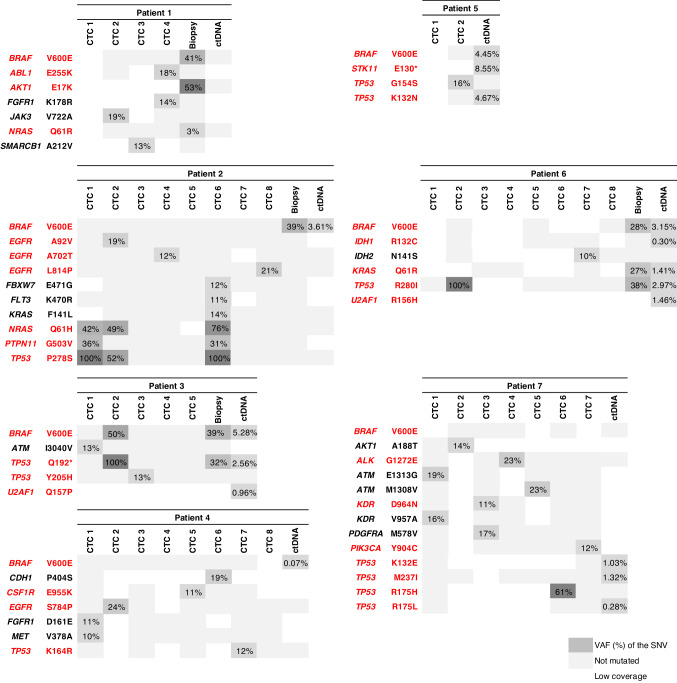
Mutational profiles of CTCs, matched tumour biopsies and cfDNA at combined dabrafenib plus trametinib therapy failure. Variants in red are known or predicted as drivers according to Cancer Genome Interpreter.

Mutations detected in single-candidate CTCs (CD45^−^/CK^+^ cells), cfDNA and matched tumour biopsies are shown in Figs. 1 and 2. In P1, three out of four CD45^−^/CK^+^ cells were mutated with a VAF > 10% (CTC-2, *JAK3*^V722A^; CTC-3, *SMARCB1*^A212V^; CTC-4, *ABL1*^E255K^ and *FGFR1*^K178R^) at 46.8 months of dabrafenib-trametinib therapy. No mutation was detected in cfDNA monitoring. Three mutations including *BRAF*^V600E^, *AKT1*^E17K^ and *NRAS*^Q61R^ were identified in the tumour biopsy performed at 55 months, when the disease progressed slowly in the lung. The *AKT1*^E17K^ mutation was also detected in tissue biopsy at baseline to dabrafenib-trametinib as previously reported [11]. In P2, at the time of PD (adrenal) after 16.9 months of treatment, five out of eight candidate CTCs were mutated (CTC-1, *NRAS*^Q61H^, *PTPN11*^G503V^, *TP53*^P278S^; CTC-2, *EGFR*^A92V^; *NRAS*^Q61H^, *TP53*^P278S^; CTC-4, *EGFR*^A702T^; CTC-6, *FBXW7*^E471G^, *FLT3*^K470R^, *KRAS*^F141L^, *NRAS*^Q61H^, *PTPN11*^G503V^, *TP53*^P278S^; CTC-8, *EGFR*^L814P^). In cfDNA and tissue biopsy sampled at PD, *BRAF*^V600E^ was the only mutation detected. In P3, *BRAF*^V600E^ and *TP53*^Q192*^ variants were detected in one candidate CTC (CTC-2) and matched cfDNA and tumour biopsy performed at multisite PD (bone, peritoneal, nodal). Two additional candidate CTCs were also mutated (CTC-1, *ATM*^I3040V^; CTC-3, *TP53*^Y205H^), while the *U2AF1*^Q157P^ mutation was found in cfDNA. Five out of eight candidate CTCs were mutated (CTC-1, *FGFR1*^D161E^, *MET*^V378A^; CTC-2, *EGFR*^S784P^; CTC-5, *CSF1R*^E955K^; CTC-6, *CDH1*^P404S^; CTC-7, *TP53*^K164R^) at PD in P4. In the cfDNA sample collected at a time point closer to that of the CTC sample, *BRAF*^V600E^ was detected at a low VAF (0.07%). One out of two candidate CTCs was mutated (CTC-2, *TP53*^G154S^) in P5 at multisite PD (liver, brain) at 7.6 months. In the cfDNA sample, *BRAF*^V600E^, *TP53*^K132N^ and *STK11*^E130*^ mutations were detected. In P6, we detected two out of eight candidate CTCs mutated (CTC-2, *TP53*^R280I^ and CTC-7, *IDH2*^N141S^) at PD (bone, liver). Concordance on the *TP53* mutation between CTCs, cfDNA and tumour biopsy was observed in this patient. Of note, *TP53* mutation was also detected in tissue biopsy at baseline treatment [11]. *BRAF*^V600E^ *and KRAS*^Q61R^ mutations were identified in both the cfDNA and tumour tissue. In P7, the seven candidate CTCs isolated from the two blood samples were mutated (CTC-1, *ATM*^E1313G^, *KDR*^V957A^; CTC-2, *AKT1*^A188T^; CTC-3, *KDR*^D964N^, *PDGFRA*^M578V^;CTC-4, *ALK*^G1272E^; CTC-5, *ATM*^M1308V^; CTC-6, *TP53*^R175H^; CTC-7, *PIK3CA*^Y904C^) after 70 months of treatment. Three *TP53* mutations (*TP53*^K132E^, *TP53*^M237I^, *TP53*^R175L^) were found in cfDNA on treatment but all were different from that detected in CTCs.

Overall, *BRAF*^V600E^ was found in 4/4 resistance tumour biopsies [VAF range, 28–41%] and in 5/7 cfDNA samples [VAF range, 0.07–5.3%]. In contrast, only 1/26 mutated CTCs (P3, CTC-2) was *BRAF*^V600E^ mutated in accordance with cfDNA and tumour tissue samples. A total of 34 *BRAF*-independent mutations were identified in single CTCs [VAF range, 10–100%], while the number of *BRAF*-independent mutations was lower in tumour biopsies (5 mutations) and cfDNA (11 mutations) bulk samples. All CTC mutations were not implicated in the MAPK pathway except P2 CTC. In most cases, CTCs had different mutational profiles. Recurrent mutations (*NRAS*^Q61H^, *PTPN11*^G503V^, *TP53*^P278S^) in two or three CTCs were only observed in P2. Six of the seven patients had *TP53* mutations with variable VAF in single CTC samples. These data evidenced a much higher mutational diversity in CTCs compared to tumour tissue biopsies and cfDNA. Moreover, they showed important intra-patient tumour heterogeneity out of the MAPK pathway, which is missed in most cases by bulk analyses. Therefore, CTCs analysed at the single-cell level capture a different tumour mutational landscape than the one detected in the tumour biopsies and cfDNA. Moreover, in contrast with tumour tissue biopsies and cfDNA, it is noteworthy that only one CTC harboured a *BRAF*^V600E^ mutation, which suggests that resistance to BRAF inhibitors may not be driven by *BRAF*^V600E^-mutated CTCs.

### CNA analysis of single CTCs and tumour biopsies

**Fig. 3:**
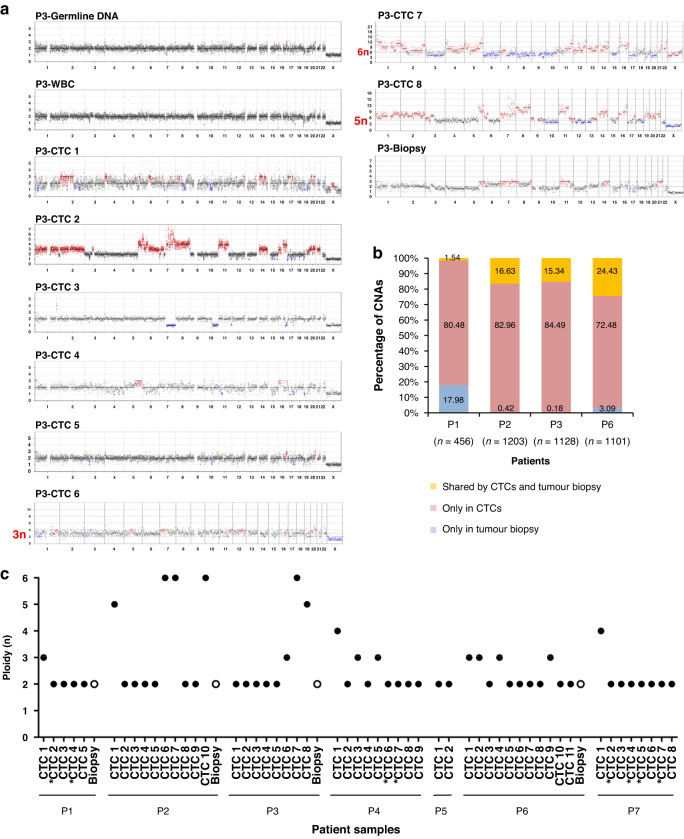
Low-pass whole-genome CNA profiles and ploidy of CTCs and matched tumour biopsies at combined dabrafenib plus trametinib therapy failure. **a** CNA profiles of CTCs, corresponding germline DNA and leucocyte controls, and matched tumour biopsies from patient P3. Gains are shown in red, losses in blue. **b** Comparative CNA analysis of CTCs and matched tumour biopsies from patients P1, P2, P3 and P6. Numbers of total CNAs detected in each patient are mentioned in parentheses. **c** Ploidy level determined for each single CTC (black dots) and tumour biopsy (white dots) samples. *CTC samples that show a flat diploid CNA profile but harbour mutations.

LP-WGS is relatively tolerant to lower WGA quality. A total of 90/144 (62.5%) candidate CTC samples were tested by LP-WGS and included 49 samples with a GII of 1 or 2, to increase the data points on selected patients. Among these 90 single-candidate CTC samples, five (with a GII of 1) did not pass the LP-WGS quality controls, 40 had flat CNA profiles and 45 (50%) presented altered CNA profiles. 8/40 candidate CTCs with flat profiles were mutated, thus confirming their tumour origin. We could not determine whether the remaining 32 candidate cells were epithelial normal cells or tumour cells without a detectable alteration. A total of 1850 CNAs (Supplementary Table 3) were identified in gain or loss regions across the 45 single CTCs and four tumour biopsies. The number of CNAs was highly variable among CTCs ([range, 13–714]; median, 102] as evidenced by CNA profiles and the detailed list of CNAs identified (Fig. 3a, Supplementary Fig. 2 and Supplementary Table 3). Although some recurrent alterations were observed, as in P3 CTCs, CTC CNA profiles were indicative of an important intra-patient genomic diversity. Percentages of CNAs detected in CTCs and corresponding tumour biopsies, and of CNAs exclusively detected in CTCs are shown in Fig. 3b. CNAs detected in both CTCs and corresponding tumour biopsies represented 1.5 %, 16.6%, 15.3% and 24.4% of total CNAs in P1, P2, P3 and P6 respectively. In contrast, the percentages of CNAs exclusively found in CTCs ranged from 72.5 to 84.5%. The number of CNAs exclusively detected in tumour biopsies is relatively low [range, 0.2% to 18%]. In the four patients, these percentages are roughly similar when we considered driver genes (Supplementary Fig. 3 and Supplementary Table 4). In three out of four patients (P2, P3 and P6), CNA drivers detected in tumour biopsies were also found in corresponding CTCs which had accumulated numerous additional alterations. These data showed that tumour biopsies exhibited limited genomic heterogeneity compared to CTCs. In most cases, CTCs with highly altered profiles exhibited a ploidy greater than two (Fig. 3a, c and Supplementary Fig. 2). Six patients had at least one CTC with a ploidy level estimation >2n. Important chromosomal instability (CIN) was observed in a fraction of CTCs, with 30% (16/53) of them having a ploidy higher than 2n. Eight CTCs with a ploidy ≥4n possibly experienced whole-genome doubling (WGD), which may impact tumour evolution. The four matched tumour biopsies showed a normal ploidy at 2n. Hierarchical clustering was further performed to evaluate sample similarity and identify recurrently altered chromosomal regions (Supplementary Fig. 4). Overall, data indicated a much higher intra- and inter-patient genomic diversity in CTCs than in corresponding tumour biopsies at resistance to BRAF inhibitors, which may strongly contribute to CIN and impact tumour adaptation to therapy.

### Mutation and CNA driver classification in altered signalling pathways

**Fig. 4:**
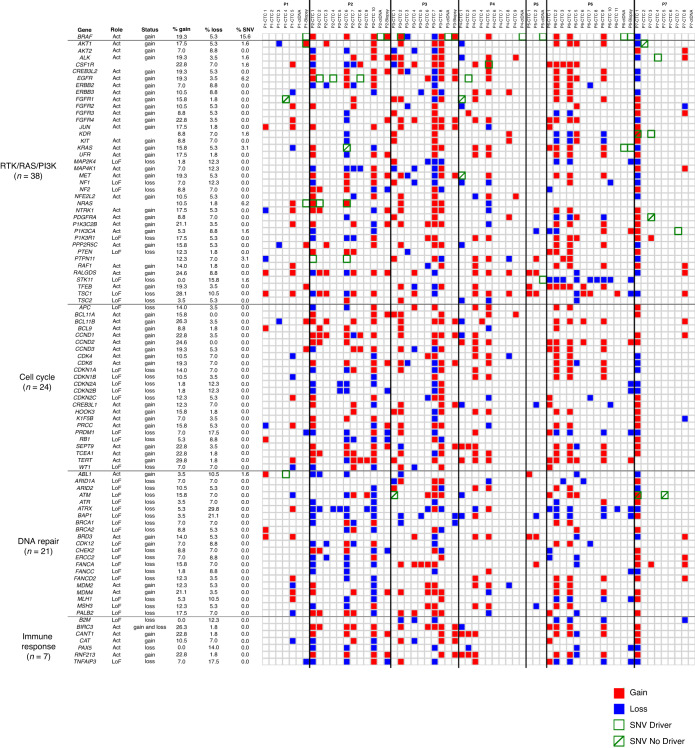
Heatmap of selected CNA and SNV oncogenic drivers in CTCs, matched tumour biopsies and cfDNA at combined dabrafenib plus trametinib therapy failure, according to their clinical relevance and/or presence in ≥5 patients. Altered genes are attributed to pathways. The four main pathways are sorted from the most altered to the least altered. The number of altered genes per pathway is shown in parentheses. CNA driver function (activating or loss of function) is mentioned in the “Role” column. Frequencies of CNAs and driver SNVs in the 57 samples (53 CTCs and 4 tumour biopsies) and the 64 samples (53 CTCs, 4 tumour biopsies and 7 cfDNA), respectively, are provided. Red and blue colours represent gains and losses, respectively.

**Fig. 5:**
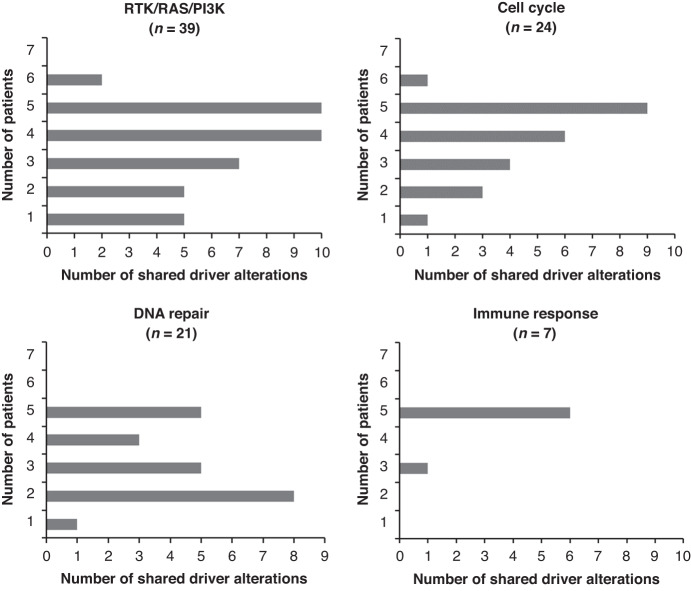
Shared recurrent driver alterations detected in the seven patients. Numbers of recurrent CNA and SNV driver alterations in RTK/RAS/PI3K, cell cycle, DNA repair and immune response pathways are presented.

Mutations and selected CNA drivers according to their clinical relevance and/or presence in at least 5/7 patients were classified in signalling pathways (Fig. 4 and Supplementary Fig. 5). RTK/RAS/PI3K, cell cycle-related, DNA repair-related, and immune response pathways were prevalent, with a total of 38, 24, 21 and 7 altered genes respectively. Gene alterations were, in most cases, a gain of function. We further examined recurrent driver alterations among these pathways (Fig. 5 and Supplementary Table 5). Driver alterations in RTK/RAS/PI3K pathways predominantly included MAPK pathway genes with gains in *BRAF, RALGDS* (RAS-related GTPase) and *RAF1* genes. Alterations in MAPK-related pathway genes with gains in *LIFR* gene (cytokine receptor gene related to ERK signalling) and *NTRK1* (neurotrophic receptor gene involved in MAPK pathway member phosphorylation) and loss in *NF1* (negative regulator of RAS signalling) were detected in CTCs of 5/7 patients. Gains in *TFEB*, *FGFR1*, *FGFR2* and *FGFR4* genes, which transduce signals to downstream pathways such as MAPK and PI3K pathways, were also predominant. Driver alterations in cell cycle-related pathways included gains in *TERT, SEPT9* (involved in cytokinesis control), and in *CCND1*, *CCND2* and *CCND3* members of the cyclin family—key regulators of the mitotic cycle—as well as in the *PRCC* gene, which acts as a regulator of cell cycle progression. *CDKN2A* loss in cell cycle-related pathways is also predominantly observed. Loss of *ATRX* and *BAP1* and gain in *MDM4* and *BRD3* involved in response to DNA damage, chromatin dynamics and TP53 activity regulation, are also recurrently observed. *BIRC3* and *PAX5* genes, involved in cell invasion, migration and metastasis among other functions were recurrently altered in immune and inflammatory response-related pathways. Overall, numerous oncogenic drivers were activated both in the MAPK- and in MAPK-independent pathways such as cell cycle-related, DNA repair and immune response, reflecting the remarkable genomic heterogeneity of CTCs at resistance to BRAF inhibition.

## Discussion

Resistance mechanisms to BRAF-targeted therapy, unlike other targeted therapies against oncogenic driver alterations (e.g., EGFR or ALK tyrosine kinase inhibitors), are poorly understood. In this work, we report for the first time, the molecular profiling of CTCs at resistance to BRAF-targeted therapy in a pilot study of patients with advanced *BRAF*^V600E^-mutant NSCLC. In seven patients with *BRAF*^V600E^ mutation, 90 single CTCs were sequenced and resistance mutations were compared to the ones detected in matched cfDNA and tumour biopsies (four patients). In contrast to tumour tissue biopsies and cfDNA, only one CTC harboured a *BRAF*^V600E^ mutation, which suggested that resistance to BRAF inhibitors was not driven by *BRAF*^V600E^-mutated CTCs in these patients. Whole-genome profiling through CNA analysis indicated that CTCs encompassed the majority of CNAs found in the corresponding tumour biopsies but had also accumulated numerous additional alterations not detected in tumour biopsies. Classification of oncogenic mutations and CNAs drivers in signalling pathways revealed the activation of MAPK and MAPK-related pathways together with that of cell cycle, DNA repair and immune response-related mechanisms. Overall, our single CTC data revealed high genomic intra- and inter-patient genomic diversity in MAPK, MAPK-related and MAPK-independent pathways at resistance to BRAF inhibition, which was missed by bulk analyses on tumour biopsies and cfDNA.

CTC counts were determined according to both the CellSearch technique and hematopoietic blood-cell depletion combined to immunofluorescence staining and FACS. This second approach offers the advantage of having no a priori on CTC phenotype. Several studies including ours have reported low counts of CTCs with epithelial characteristics in NSCLC, even at an advanced stage. We and others have shown that larger CTC numbers can be identified using various non-EpCAM-based detection methods, most likely because CTCs that have lost their epithelial features and express epithelial-to-mesenchymal transition (EMT) markers can be missed by the CellSearch [31–33]. In *ALK*-positive patients, we previously reported that *ALK*-rearranged CTCs may express EMT characteristics [18, 27]. As expected, according to our non-EpCAM-based detection method, higher numbers of candidate CTCs were detected and isolated as single cells whose tumour origin was confirmed by mutational and low-pass whole-genome sequencing.

A striking result is that only one CTC among 26 mutated CTCs carried the *BRAF*^V600E^ mutation whereas the 4/4 tumour biopsies and 5/7 cfDNA samples were *BRAF*^V600E^-positive. In contrast, multiple BRAF-independent mutations, mainly out of the MAPK pathway were detected in single CTCs by targeted PCR/NGS, while the number of these mutations was much lower in tumour biopsies and cfDNA samples. In most cases, high intra- and inter-patient diversity in driver mutations was observed in CTCs compared to tumour tissue biopsies and cfDNA. Using whole-genome profiling, we also detected multiple gains and, to a lesser extent, losses that were not observed in matched tumour tissue. Interestingly, in the four patients for whom the tumour biopsy was available at resistance to therapy, CTCs recapitulated the CNA profile of the matched tumour tissue, while revealing important additional diversity in CNA drivers. Furthermore, 30% of CTC clones presented a ploidy level estimation indicative of important CIN, while matched tumour biopsies had a normal ploidy. Therefore, CTCs revealed a unique genomic representation of resistance that may be of clinical relevance and complementary to data provided by tumour tissue and cfDNA, which contributed similar information. In contrast to cfDNA, which is mainly released from apoptotic or necrotic tumour cells, CTCs are living cells that are amenable to detailed genomic analysis at the single-cell level, and thus can provide new insight into the biology and vulnerabilities of metastatic cancer. They likely represent dynamic aggressive cell clones that are replenished from different metastatic sources and may be highly relevant in metastatic progression. By providing a snapshot evaluation of tumour heterogeneity, CTCs may therefore offer a unique benefit as a liquid biopsy component to help improving our understanding of therapeutic resistance.

We further investigate cancer-related pathways that could be associated with dabrafenib +/- trametinib resistance. For this purpose, we selected a total of 141 genes of greatest relevance, according to their presence in most of the patients (at least 5 out of 7) and/or according to their clinical interest as key oncogenic pathways in NSCLC. We observed an extensive diversity involving different mechanisms (e.g., MAPK, RTK/PI3K, cell cycle, DNA repair or immune response), with a high rate of CNAs (gain/loss) and potential mutations in key driver genes. This suggests that intra- and inter-patient tumour heterogeneity may be a critical feature in resistance to BRAF inhibition. Based on data available, two mechanisms of acquired BRAF resistance have been described: MAPK-dependent, related to the reactivation of the MAPK pathway, and MAPK-independent mechanisms, though alternative pathways [34]. So far, studies carried out in tumour biopsies and cfDNA have reported that alterations in MAPK pathway are predominant both in melanoma [34] and NSCLC [10, 11, 16] at BRAF-targeted therapy failure. Here, CNAs in MAPK pathway were found in six out of seven patients (86%), leading to the reactivation of *ERK* independently of RAS [35]. We identified *BRAF* copy number gains in four out of seven patient CTCs and losses in two patients. This is higher compared to data on melanoma (8–20%) [36], and no previous data in lung cancer has been reported. The presence of multiple CNAs in *KRAS, NRAS* or *NF1* and others genes of the MAPK pathway identified in CTCs are indicative of increased activity of this molecular pathway at *BRAF* resistance. Dysregulations in PI3K and AKT signalling have also been linked to BRAF inhibitor resistance in melanoma [37]. Here, we identified PI3K/AKT pathway signalling altered in 5/7 patients (predominantly gains). Although PI3K/AKT signalling pathway has been described as an alternative pathway in BRAF resistance, only P5 had PI3K/AKT signalling alterations with no concomitant MAPK alterations, which questions the potential role of PI3K/AKT in resistance in our study. We also observed CNA in *EGFR*, and other *HER* family genes, as well as in *FGFR1/2* genes, concomitantly with *EGFR* mutations in P3 and P7. DNA repair signalling alterations were found in most patients, including clinically relevant genes such as *ATM/ATR* or *BRCA1/2* with concomitant *ATM* mutations detected in two cases (P3, P7). Immune signalling pathways were also implicated, suggesting promising data that could lead new treatment options in the future. We did not observe any relevant difference on cancer pathways signalling according to the clinical profile, type of treatment (dabrafenib vs. dabrafenib + trametinib), or duration of response, consistent with previous data observed in tissue and ctDNA cohorts [11, 16]. However, considering the sample size, these clinical observations should be further explored in larger cohorts. As we previously reported in ALK-positive patient CTCs at resistance to ALK inhibitors [26], we observed here that several alterations may occur within a single CTC in *BRAF*^V600E^ NSCLC at therapeutic resistance. Our data show the heterogeneity of resistance with an entangled scenario of pathways signalling altered which could likely be related to our single-cell data and the unique nature of CTCs.

Unfortunately, there is no approved targeted therapy at failure to treatment with dabrafenib plus trametinib [38]. A better understanding of resistance mechanisms to these therapies is therefore crucial for the development of more effective therapeutic strategies in this patient population [8]. New targeted therapies are currently under clinical development, including ERK1/2 inhibitors such as ulixertinib (NCT04566393) or LY3214996 (NCT02857270), combined or not with other BRAF-targeted therapies, to overcome acquired resistance related to the reactivation of ERK signalling through MAPK pathway [39]. Although exploratory, our study may provide clinically relevant information on alternative signalling pathways involved in BRAF^V600E^ resistance that could play a role on the future development of molecular therapeutic strategy. Therapeutic opportunities that could be considered in this complex context include for example, the addition of ATM/ATR inhibitors under development to target DNA repair alterations, or immune checkpoint inhibitors that may contribute to enhance T-cell responses previously induced with BRAF inhibitors [40]. Indeed, recent data suggest that BRAF-targeted therapy may foster host immune responses to melanoma, characterised by enhanced expression of melanoma differentiation antigens, reduced levels of immunosuppressive cytokines in the microenvironment, a CD8 T-cell response and T-cell-mediated cytotoxicity [41]. Other options include therapies targeting oncogenic drivers (e.g., *KRAS*, *or HER* family, *FGFR1/2,* etc.) such as novel antibody-drug conjugates (ADC) agents. Nevertheless, these hypotheses remain to be firstly evaluated in preclinical functional studies.

The main limitations of this study included the small sample size of this pilot study, the lack of tissue biopsies to be compared to CTCs in 3 cases and different techniques for CTC/cfDNA/tissue sequencing with different limits of detection. Thirty-two candidate CTCs, among 90 sequenced, harboured a flat CNA profile with no mutation detected by the targeted panel. The technique may allow to capture normal epithelial cells, as previously reported [42]. Usually, these cells represent a minor population, as we previously observed [27]. In our present study, these cells are present in relatively high proportions. This might result from the treatment, the disease or a technical reason during single-cell isolation by FACS. Furthermore, our study is limited to a single time point (disease progression) and does not allow us to monitor a longitudinal change in the mutational profile during treatment which correlates to the clinical profile of resistance. However, we detected alterations in CTCs that were not detected in the matched single-site tumour biopsy, including numerous alterations in MAPK and alternative pathways, with potential relevance for clinical development (e.g., *ATM*, *ATR*, etc.). Finally, it is important to emphasise the unique characteristics of this study population. We observed a median PFS that exceeded the outcomes reported in previous studies [5, 6], which suggests that our cohort may represent a subpopulation particularly responsive to the BRAF inhibitors. Thus, the outcomes observed in this study may not be generalisable to a patient population where resistance may emerge earlier. To our knowledge, no previous studies have been conducted to characterise CTCs at BRAF treatment failure in NSCLC *BRAF-*mutant population. Our analysis at the single-cell level provides unique insight into the heterogeneous mechanisms of resistance to BRAF inhibitors, revealing both MAPK-independent and MAPK-dependent pathways and a genetic diversity of CTCs that can be clinically informative.

Despite these limitations, single CTC sequencing is a helpful tool to inform drug resistance mechanisms and provide in-depth characterisation of the genomic landscape of resistant cell clones to target in *BRAF*^V600E^-mutant NSCLC. This information, integrated with cfDNA analysis, provides perspectives that may be of great utility to clinicians in order to guide precision medicine at BRAF inhibition progression and contribute to the development of new therapeutic strategies. Finally, the clinical relevance of heterogeneity is yet to be defined by demonstrating how it can guide therapy and affect patient outcomes.

### Supplementary information

Mezquita and Oulhen et al_Supplementary info

Supplementary Information Figure file

Supplementary Table 1

Supplementary Table 2

Supplementary Table 4

Supplementary Table 3

Supplementary Table 5

## Data Availability

All the data supporting the findings of this study are available within the article and its supplementary information files and from the corresponding authors upon reasonable request.
