# Supplementary material for: Resistance to BRAF inhibition explored through single circulating tumour cell molecular profiling in BRAF-mutant non-small-cell lung cancer
Source: Br J Cancer. 2024 Jan 4;130(4):682–93. doi: 10.1038/s41416-023-02535-0 (PMC10876548; doi:10.1038/s41416-023-02535-0)
Supplement: Supplementary file 1 — Mezquita and Oulhen et al_Supplementary info [file 41416_2023_2535_MOESM1_ESM.docx]

**Resistance to BRAF inhibition explored through Single Circulating Tumor Cell molecular profiling in *BRAF-*mutant Non-Small-Cell Lung Cancer**

Laura Mezquita^1,2*^, Marianne Oulhen^3,4*^, Agathe Aberlenc^3,4^, Marc Deloger^5^, Mihaela Aldea^1^, Aurélie Honore^6^, Yann Lecluse^7^, Karen Howarth^8^, Luc Friboulet^4^, Benjamin Besse^1^, David Planchard^1$^, Françoise Farace^3,4$#^

*^1^ Gustave Roussy, Université Paris-Saclay, Department of Medicine, F-94805, Villejuif, France*

*^2^ Medical Oncology Department, Hospital Clinic of Barcelona, Laboratory of Translational Genomics and Targeted Therapies in Solid Tumors, IDIBAPS, Barcelona, Spain*

*^3^ Gustave Roussy, Université Paris-Saclay, “Rare Circulating Cells” Translational Platform, CNRS UMS3655 – INSERM US23 AMMICA, F-94805, Villejuif, France*

*^4^ INSERM, U981 “Identification of Molecular Predictors and new Targets for Cancer Treatment”, F-94805, Villejuif, France*

*^5^ Gustave Roussy, Université Paris-Saclay, Bioinformatics Platform, CNRS UMS3655 – INSERM US23 AMMICA, F-94805, Villejuif, France*

*^6^ Gustave Roussy, Université Paris-Saclay, Genomic Platform, CNRS UMS3655 – INSERM US23 AMMICA, F-94805, Villejuif, France*

*^7^ Gustave Roussy, Université Paris-Saclay, “Flow cytometry and Imaging” Platform, CNRS UMS3655 – INSERM US23AMMICA, F-94805, Villejuif, France*

*^8^ Inivata Ltd, Granta Park, Cambridge, United Kingdom*

^*^ LM and MO contributed equally to the study

^$^ DP and FF contributed equally to the study

^#^ **Corresponding author:** Dr Françoise Farace, Gustave Roussy, Université Paris-Saclay, “Rare Circulating Cells” Translational Platform, CNRS UMS3655 – INSERM US23 AMMICA, F-94805, VILLEJUIF France Phone: (33)142115198.

E­mail: [francoise.farace@gustaveroussy.fr](mailto:francoise.farace@gustaveroussy.fr)

**Supplementary Methods**

**Isolation of genomic DNA from blood and tumor biopsies**

DNA from formalin-fixed paraffin-embedded tumor biopsies was purified with QIAamp DNA FFPE Tissue kit (Qiagen) according to manufacturer’s protocol. Germline control DNA was purified from whole-blood samples with DNeasy blood and tissue kit (Qiagen). 1 ng of germline control DNA and genomic DNA from biopsies were amplified using the Ampli1 WGA kit (Menarini Silicon Biosystems) only for the genome copy-number profiling by low-pass whole genome sequencing (LP-WGS).

**CfDNA isolation and analysis**

Blood samples (20mL) were prospectively collected in Streck tubes (Cell-Free DNA BCT^®^) for cfDNA monitoring. cfDNA analyses were conducted by Inivata (Cambridge, UK) and Research Triangle Park (NC, US) using InVisionFirst^®^-Lung, which identifies single-nucleotide variants, insertions and deletions with whole gene and gene hotspots across a 36-gene panel.

**Tissue DNA sequencing**

In four patients, tissue samples were analyzed using targeted NGS with the Ion Torrent PGM (ThermoFisher Scientific) sequencer, which includes exons containing molecular hotspots of 82 cancer-related genes (Mosc4 customized panel developed with Ion AmpliSeq custom design). Biopsy samples required a tumor cell content ≥ 10% for undergoing NGS (1).

**Bioinformatic workflow for targeted NGS**

Sequence reads were trimmed for Ampli1 adapters with Cutadapt (v1.14) (2) and the following parameters: “-a ACTGACAGCAGGAATCCCACT -g AGTGGGATTCCTGCTGTCAGT -n 2 -e 0.2”. Reads were then aligned to the human genome build hg38/GRCh38.p7 using the Burrows–Wheeler Aligner (BWA v0.7.15-r1140) tool (3) with the following parameters: “mem -R '@rg\tID:GRCh38\tSM:{sample}\tPL:Illumina'”. Duplicated reads were marked but not removed using Sambamba (v0.6.5) markdup (4). Variant calling of single-nucleotide variants (SNV) and small insertions/deletions was performed using the Broad Institute's GATK (v3.7) Haplotype Caller in GVCF mode for germline variants following the steps described in the GATK3 best practices : GenotypeGVCFs, SelectVariants (-selectType SNP) and VariantFiltration (--filterExpression "QD < 2.0 || FS > 60.0 || MQ < 40.0 || MQRankSum < -12.5 || ReadPosRankSum < -8.0"). MuTect2 tool (v2.0,--output_mode EMIT_VARIANTS_ONLY --max_alt_alleles_in_normal_count 2 --max_alt_allele_in_normal_fraction 0.04 --maxReadsInRegionPerSample 100000) was used for somatic variants (5–7).

Ensembl's Variant Effect Predictor (VEP, release 87, homo_sapiens_refseq_vep_87_GRCh38) was used to annotate variants with respect to functional consequences (type of mutation and prediction of the functional impact on the protein by SIFT.2.2 and PolyPhen 2.2.2) and frequencies in public (dbSNP147, 1000Genomes phase 3, ExAC r3.0, COSMIC v79) (8). To rescue variants not detected by Mutect2, we performed a simple base counting using bam-readcount (v0.8) (https://github.com/genome/bam-readcount; parameters -q 20 -b 20 –w 1). We selected variants with a sequencing depth >=50, >=10 mutated reads, and a variant allele fraction (VAF) >3% if sample is a pool of CTCs or >10% if sample is a single CTC, and we removed variants detected in matched germline DNA or in >=2 unrelated germline DNAs.

**Bioinformatic workflow for LP-WGS**

The workflow of the bioinformatics analysis was performed by Menarini Silicon Biosystems.

**Sequence alignment**

The obtained FASTQ files were aligned to the hg19 human reference sequence using Burrows-Wheeler Aligner version 0.7.12 (BWA) using mem algorithm with default parameters (3).

**Copy number alterations (CNA) calling and ploidy determination**

CNAs in the data were identified using Control-FREEC software (version 11.0) with control-free mode and coefficient of variation parameter set to 0.06 (9). Ploidy level was automatically estimated by Menarini Silicon Biosystems pipeline for each library based on best fitting of profiles to underlying copy number levels with different ploidies ranging from 2 to 6 (10). For each ploidy, the root mean square error (RMSE) and percentage of genome explained are calculated using Control-FREEC. Ploidy is selected based on minimization of RMSE and maximization of percentage of genome explained. All the other Control-FREEC parameters were set to default values.

**Hierarchical clustering**

For clustering analysis, starting from the median ratio obtained by the Control-FREEC software, each profile was normalized on fixed bins length (weighted mean on 250,000 bp windows) and log2 values were calculated. Hierarchical clustering was performed using the “euclidean” distance metric and “ward” clustering method. The heatmap shows the log2 values between −2 and +2 with positive values in red (gain) and negative values in blue (loss). Copy-number profile plots and clustering heatmaps were automatically generated by Menarini Silicon Biosystems pipeline.

**Supplementary References**

1. Recondo G, Mahjoubi L, Maillard A, Loriot Y, Bigot L, Facchinetti F, et al. Feasibility and first reports of the MATCH-R repeated biopsy trial at Gustave Roussy. NPJ Precis Oncol [Internet]. 2020 Sep 8 [cited 2023 Mar 13];4. Available from: http://www.ncbi.nlm.nih.gov/pmc/articles/PMC7478969/

2. Martin M. Cutadapt removes adapter sequences from high-throughput sequencing reads. EMBnet.journal. 2011 May 2;17(1):10–2.

3. Li H, Durbin R. Fast and accurate short read alignment with Burrows–Wheeler transform. Bioinformatics. 2009 Jul 15;25(14):1754–60.

4. Tarasov A, Vilella AJ, Cuppen E, Nijman IJ, Prins P. Sambamba: fast processing of NGS alignment formats. Bioinformatics. 2015 Jun 15;31(12):2032–4.

5. DePristo MA, Banks E, Poplin RE, Garimella KV, Maguire JR, Hartl C, et al. A framework for variation discovery and genotyping using next-generation DNA sequencing data. Nat Genet. 2011 May;43(5):491–8.

6. Cibulskis K, Lawrence MS, Carter SL, Sivachenko A, Jaffe D, Sougnez C, et al. Sensitive detection of somatic point mutations in impure and heterogeneous cancer samples. Nat Biotechnol. 2013 Mar;31(3):213–9.

7. Van der Auwera GA, Carneiro MO, Hartl C, Poplin R, del Angel G, Levy-Moonshine A, et al. From FastQ data to high confidence variant calls: the Genome Analysis Toolkit best practices pipeline. Curr Protoc Bioinforma Ed Board Andreas Baxevanis Al. 2013 Oct 15;11(1110):11.10.1-11.10.33.

8. McLaren W, Gil L, Hunt SE, Riat HS, Ritchie GRS, Thormann A, et al. The Ensembl Variant Effect Predictor. Genome Biol [Internet]. 2016 Jun 6 [cited 2023 Mar 14];17. Available from: http://www.ncbi.nlm.nih.gov/pmc/articles/PMC4893825/

9. Boeva V, Popova T, Bleakley K, Chiche P, Cappo J, Schleiermacher G, et al. Control-FREEC: a tool for assessing copy number and allelic content using next-generation sequencing data. Bioinformatics. 2012 Feb 1;28(3):423–5.

10. Ferrarini A, Forcato C, Buson G, Tononi P, del Monaco V, Terracciano M, et al. A streamlined workflow for single-cells genome-wide copy-number profiling by low-pass sequencing of LM-PCR whole-genome amplification products. PLoS ONE [Internet]. 2018 Mar 1 [cited 2023 Mar 13];13(3). Available from: http://www.ncbi.nlm.nih.gov/pmc/articles/PMC5832318/

**Supplementary Figure and Table Legends**

**Supplementary Figure 1.** Whole genome amplification (WGA) quality controls of isolated single CTC samples. **A**. WGA quality controls of 144/150 single CTCs (96%). **B**. 60/144 single CTCs (41.7%) with GII ≥ 2 were selected for targeted NGS. **C**. 90/144 single CTCs (62.5%) with GII ≥ 1 were selected for low-pass whole genome sequencing.

**Supplementary Figure 2.** Low-pass whole genome CNA profiles of CTCs at disease progression on BRAF inhibition therapy, matched germline DNA, leucocytes controls, and tumor biopsies when available, from patients P1, P2, P4, P5, P6 and P7.

*: CTC samples that show a flat diploid CNA profile but harbor mutations.

**Supplementary Figure 3.** Comparative CNA driver analysis of CTCs and matched tumor biopsies from patients P1, P2, P3 and P6 at disease progression under BRAF inhibitor therapy. Counts of CNA drivers detected per patient are mentioned in parentheses.

**Supplementary Figure 4.** Hierarchical clustering of CTCs and matched tumor biopsies according to low-pass whole genome CNA at disease progression on BRAF inhibition therapy. The heatmap shows the log2 values between −2 and +2, with positive values in red (gain) and negative values in blue (loss) according to the main ploidy.

**Supplementary Figure 5.** Heatmap of selected CNA and SNV oncogenic drivers in CTCs, matched tumor biopsies and ctDNA at disease progression on BRAF inhibition therapy, according to their clinical relevance and/or presence in ≥ 5 patients. Altered genes are attributed to pathways. The eight remaining pathways (chromatin organization, signal transduction, apoptosis, metabolism, development, MYC, cell adhesion and RNA translation pathways) are sorted from the most altered to the least altered. The number of altered genes per pathway is shown in parentheses. CNA driver function (activating or loss of function) is mentioned in the “Role” column. Frequencies of CNAs and driver SNVs in the 57 samples (53 CTCs and 4 tumor biopsies) and the 64 samples (53 CTCs, 4 tumor biopsies and 7 cfDNA) respectively are provided. Red and blue colors represent gains and losses respectively.

**Supplementary Figure 6.** Recurrent CNA and SNV driver alterations in chromatin organization, signal transduction, apoptosis, metabolism, development, MYC, cell adhesion and RNA translation pathways in the seven patients.

**Supplementary Table 1.** Validation of targeted NGS workflow on COLO 205 cells harboring the *BRAF*^V600E^ mutation.

**Supplementary Table 2.** Whole genome amplification, targeted sequencing and low-pass whole genome sequencing quality controls of the 53 single CTCs harboring mutations and/or aberrant CNA profiles.

**Supplementary Table 3.** Detailed list of CNAs identified in 49 tumor samples (45 single CTC samples with altered profiles and four tumor biopsies).

**Supplementary Table 4.** CNA drivers common to CTCs and matched tumor biopsies in patients P2, P3 and P6.

**Supplementary Table 5.** Selection of CNA and SNV driver alterations that are clinically relevant and/or present in ≥ 5 patients for pathway classification, corresponding to 141 genes.
