# Supplementary material for: Resistance to BRAF inhibition explored through single circulating tumour cell molecular profiling in BRAF-mutant non-small-cell lung cancer
Source: Br J Cancer. 2024 Jan 4;130(4):682–93. doi: 10.1038/s41416-023-02535-0 (PMC10876548; doi:10.1038/s41416-023-02535-0)

# Supplementary Figure 1

**A.**

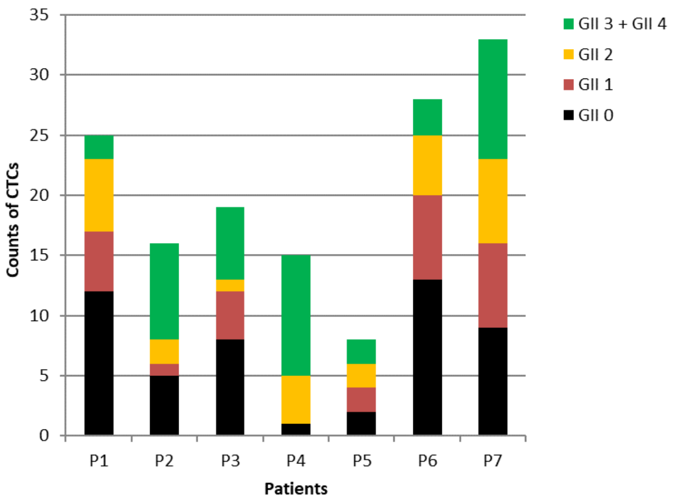


**B.**

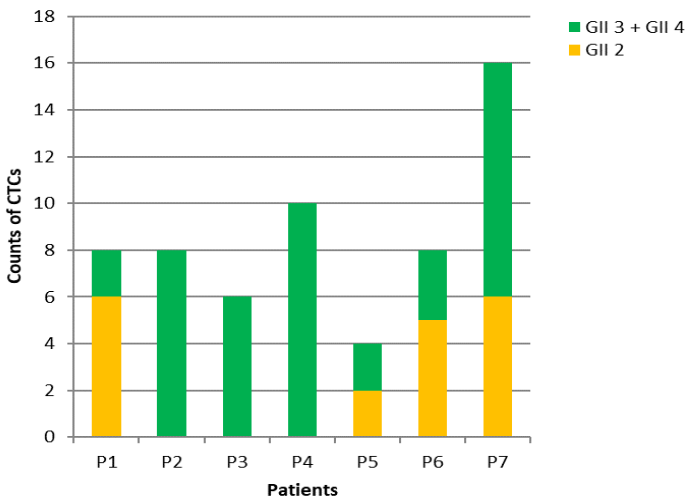


**C.**

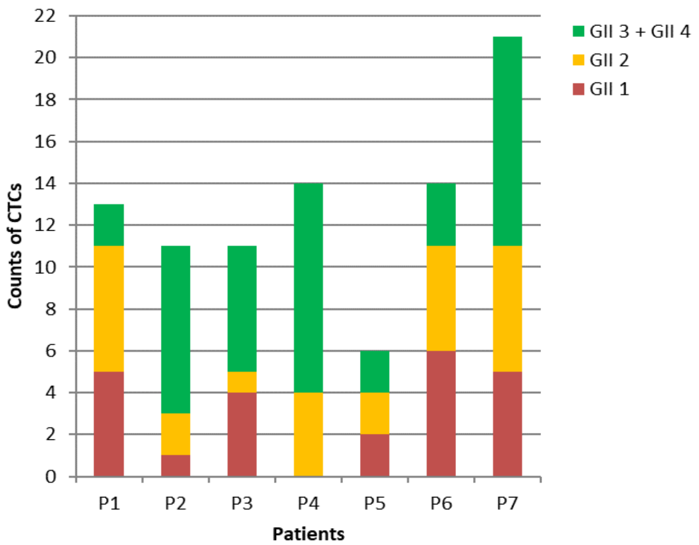

# Supplementary Figure 2

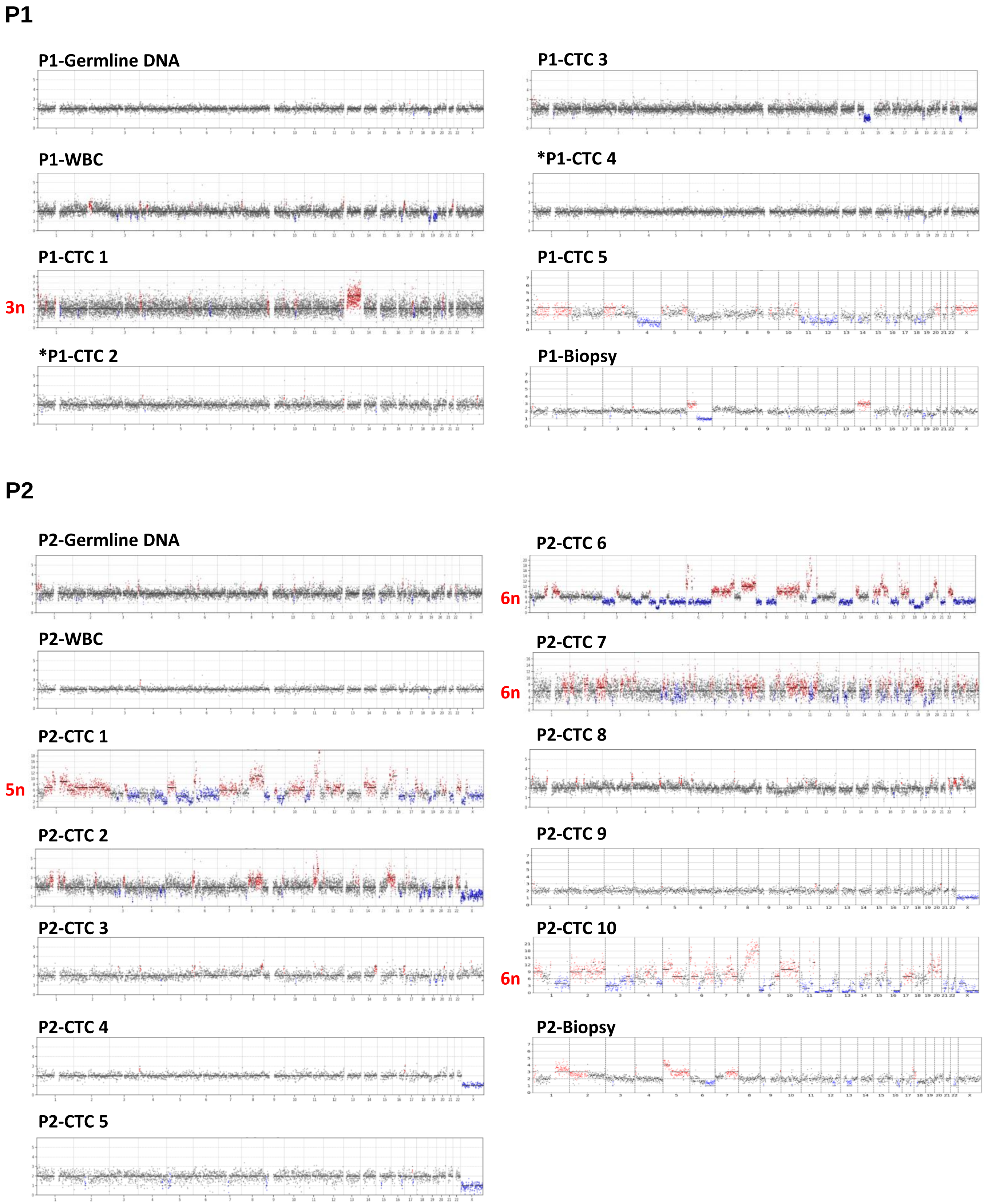

# Supplementary Figure 2

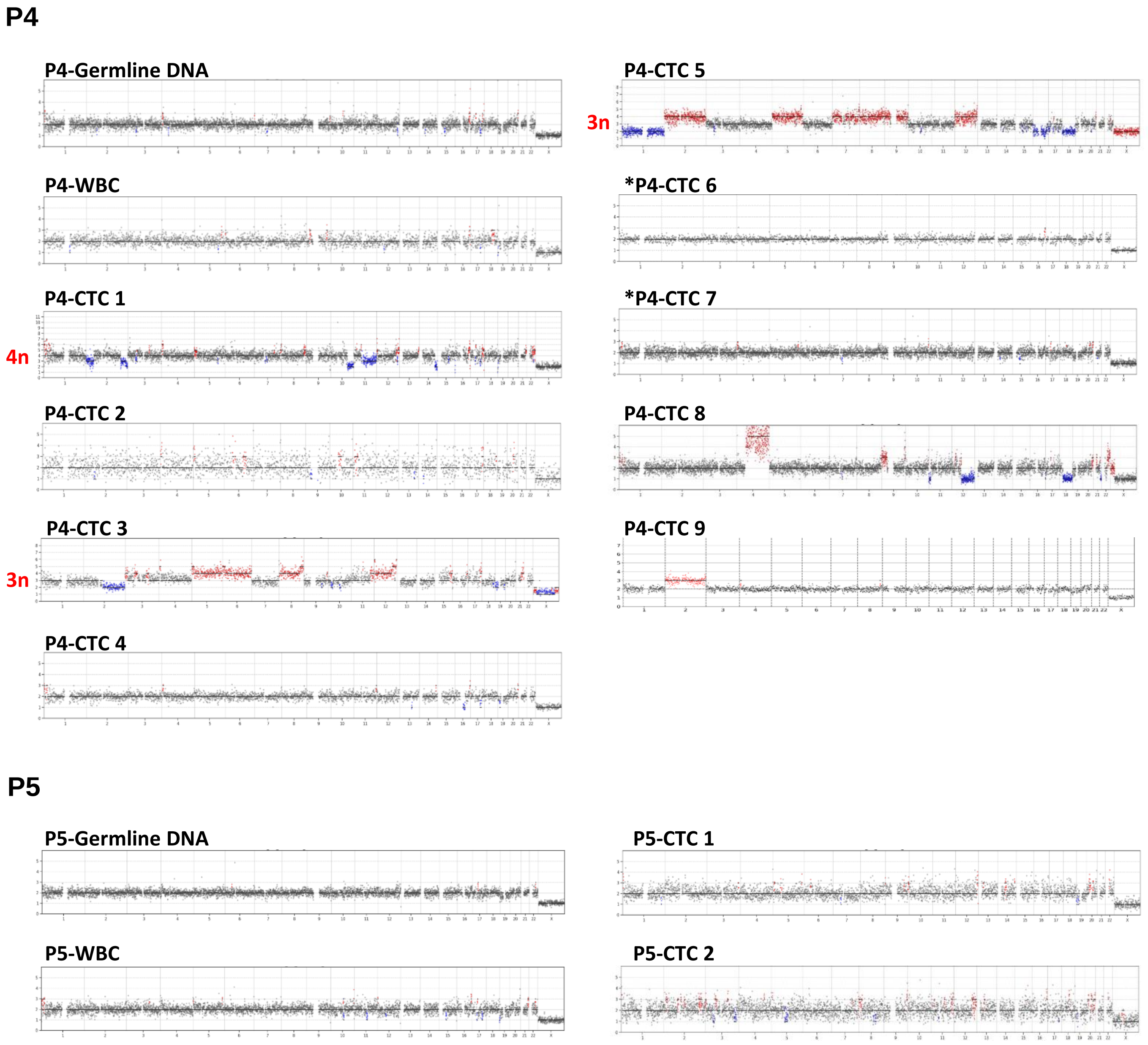

# Supplementary Figure 2

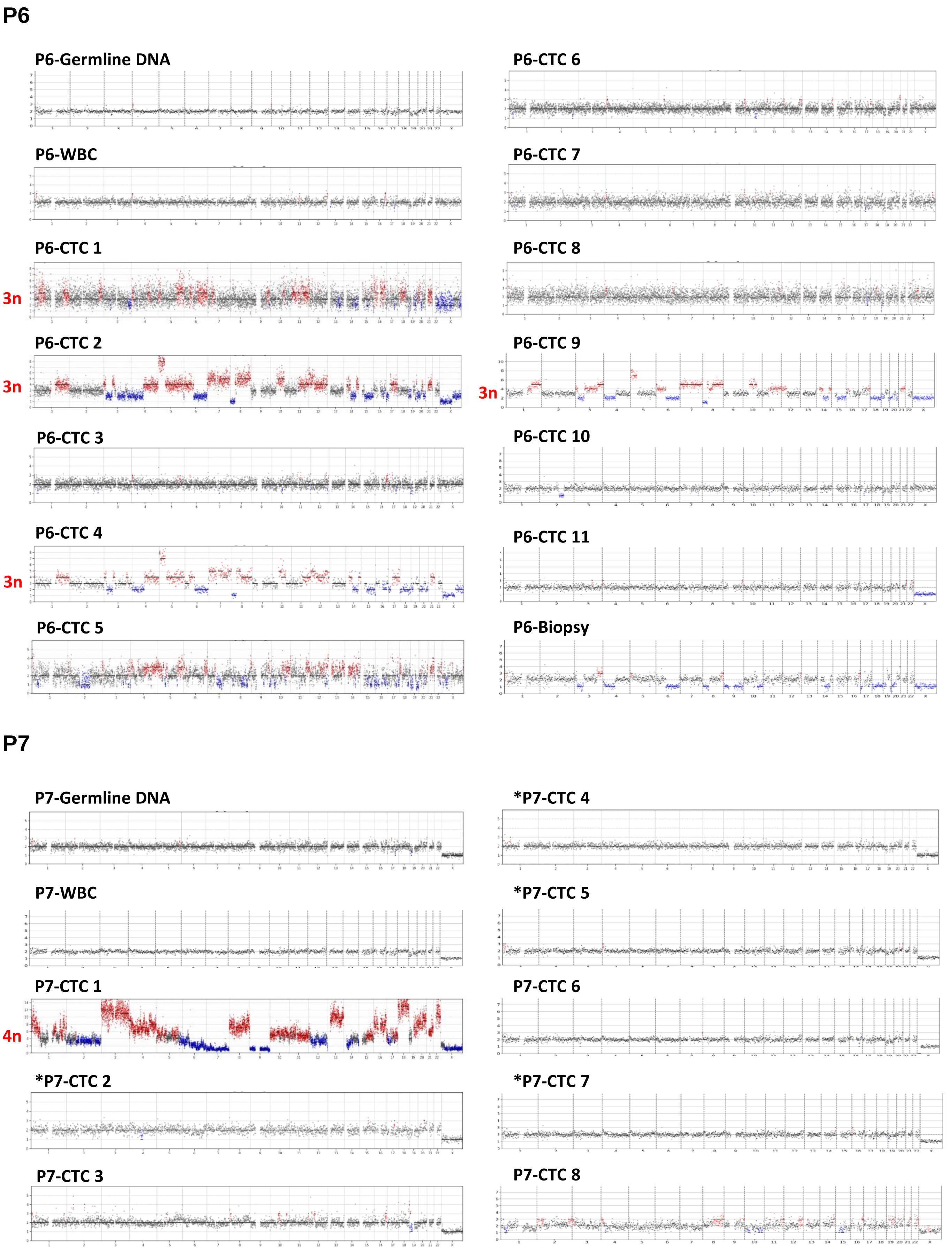

# Supplementary Figure 3

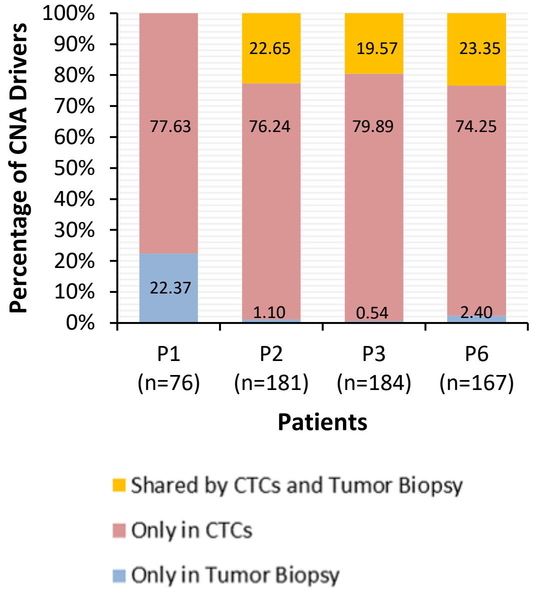

# Supplementary Figure 4

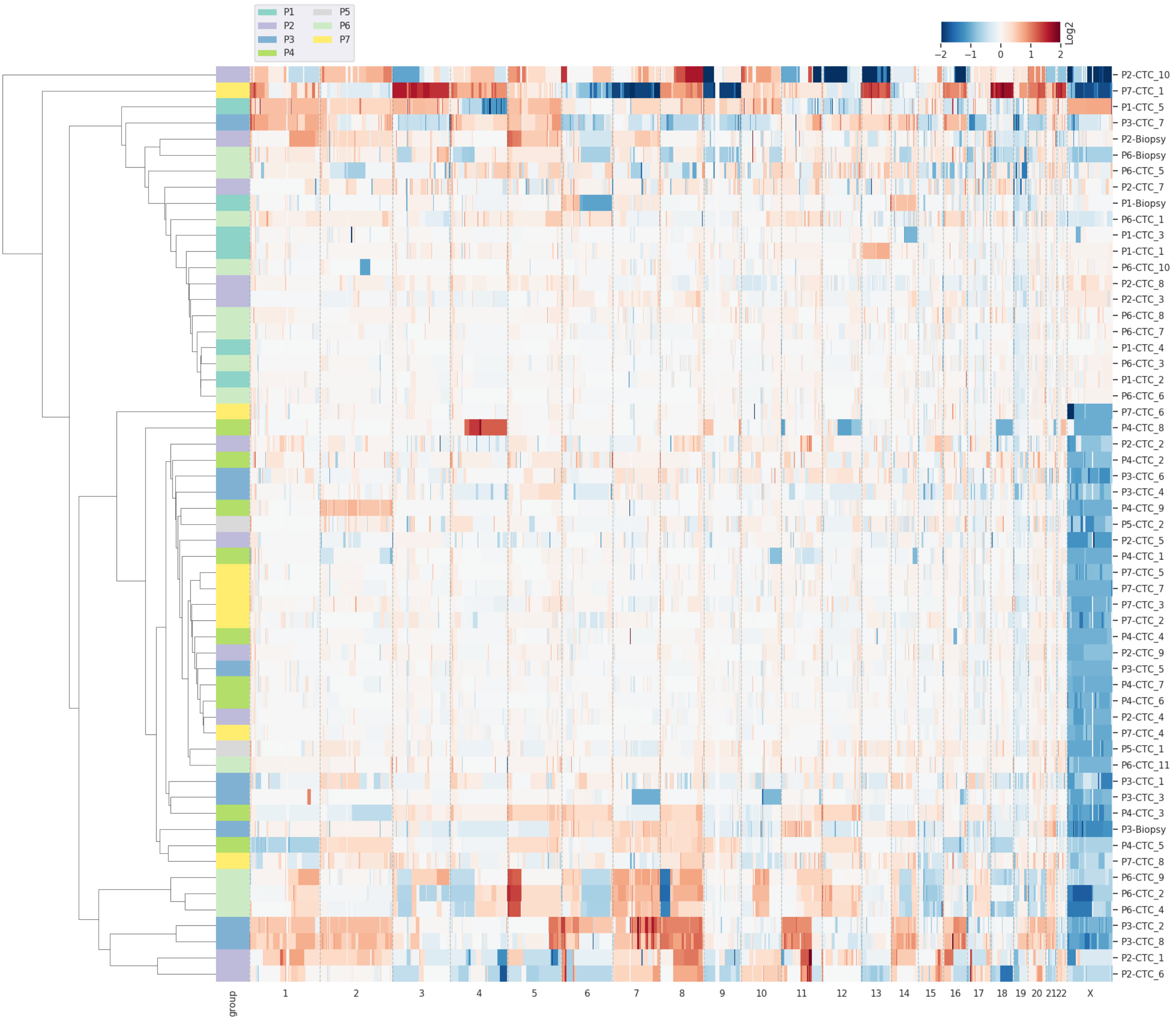

# Supplementary Figure 5

| Category | Gene | Role | Status | % gain | % loss | % SNV |
|---|---|---|---|---|---|---|
| Chromatin organization (n=13) | *BCL7A* | Act | gain | 8.8 | 8.8 | 0.0 |
| | *CHD6* | Act | gain | 22.8 | 1.8 | 0.0 |
| | *H3F3A* | Act | gain | 22.8 | 7.0 | 0.0 |
| | *KDM5A* | Act | gain | 8.8 | 3.5 | 0.0 |
| | *KDM6A* | LoF | loss | 3.5 | 29.8 | 0.0 |
| | *MEN1* | LoF | loss | 12.3 | 7.0 | 0.0 |
| | *NSD1* | Act | gain | 22.8 | 3.5 | 0.0 |
| | *PBRM1* | LoF | loss | 3.5 | 21.1 | 0.0 |
| | *SETD2* | LoF | loss | 1.8 | 21.1 | 0.0 |
| | *SETDB1* | Act | gain | 8.8 | 5.3 | 0.0 |
| | *SMARCA4* | LoF | loss | 0.0 | 28.1 | 0.0 |
| | *SMARCB1* | LoF | loss | 12.3 | 5.3 | 0.0 |
| | *WHSC1L1* | Act | gain | 14.0 | 3.5 | 0.0 |
| Signal transduction (n=9) | *ARHGEF12* | Act | gain | 22.8 | 7.0 | 0.0 |
| | *BCL3* | Act | gain | 7.0 | 8.8 | 0.0 |
| | *CBL* | Act | gain | 21.1 | 7.0 | 0.0 |
| | *EWSR1* | Act | gain | 8.8 | 5.3 | 0.0 |
| | *GNAS* | Act | gain | 22.8 | 5.3 | 0.0 |
| | *HSP90AB1* | Act | gain | 15.8 | 5.3 | 0.0 |
| | *HSPA8* | Act | gain | 21.1 | 7.0 | 0.0 |
| | *SMAD3* | LoF | loss | 3.5 | 14.0 | 0.0 |
| | *SMAD4* | LoF | loss | 5.3 | 12.3 | 0.0 |
| Apoptosis (n=8) | *BCL2* | Act | gain | 3.5 | 14.0 | 0.0 |
| | *BCL6* | Act | gain | 8.8 | 7.0 | 0.0 |
| | *NDRG1* | Act | gain | 24.6 | 0.0 | 0.0 |
| | *SET* | Act | gain | 1.8 | 14.0 | 0.0 |
| | *TP53* | LoF | loss | 3.5 | 15.8 | 23.4 |
| | *TP53BP1* | LoF | loss | 0.0 | 14.0 | 0.0 |
| | *VHL* | LoF | loss | 14.0 | 3.5 | 0.0 |
| | *ZNF292* | LoF | loss | 5.3 | 15.8 | 0.0 |
| Metabolism (n=7) | *CHCHD7* | Act | gain | 24.6 | 1.8 | 0.0 |
| | *COX6C* | Act | gain | 24.6 | 3.5 | 0.0 |
| | *IDH1* | | | 14.0 | 5.3 | 1.6 |
| | *IDH2* | Act | gain | 14.0 | 5.3 | 0.0 |
| | *NCOA2* | Act | gain | 22.8 | 1.8 | 0.0 |
| | *PPARG* | Act | gain | 15.8 | 5.3 | 0.0 |
| | *TRERF1* | Act | gain | 17.5 | 5.3 | 0.0 |
| Development (n=5) | *MITF* | Act | gain | 12.3 | 5.3 | 0.0 |
| | *PLAG1* | Act | gain | 24.6 | 1.8 | 0.0 |
| | *PLXNA1* | Act | gain | 12.3 | 5.3 | 0.0 |
| | *SOX17* | Act | gain | 22.8 | 1.8 | 0.0 |
| | *ZFHX3* | LoF | loss | 10.5 | 21.1 | 0.0 |
| MYC (n=4) | *CSDE1* | LoF | loss | 10.5 | 1.8 | 0.0 |
| | *MGA* | LoF | loss | 0.0 | 15.8 | 0.0 |
| | *MYC* | Act | gain | 29.8 | 1.8 | 0.0 |
| | *MYCN* | Act | gain | 21.1 | 1.8 | 0.0 |
| Cell adhesion (n=3) | *EPHA2* | LoF | gain | 21.1 | 1.8 | 0.0 |
| | *MACF1* | Act | gain | 12.3 | 7.0 | 0.0 |
| | *STARD13* | Act | gain | 10.5 | 5.3 | 0.0 |
| RNA translation (n=1) | *U2AF1* | | | 21.1 | 3.5 | 3.1 |

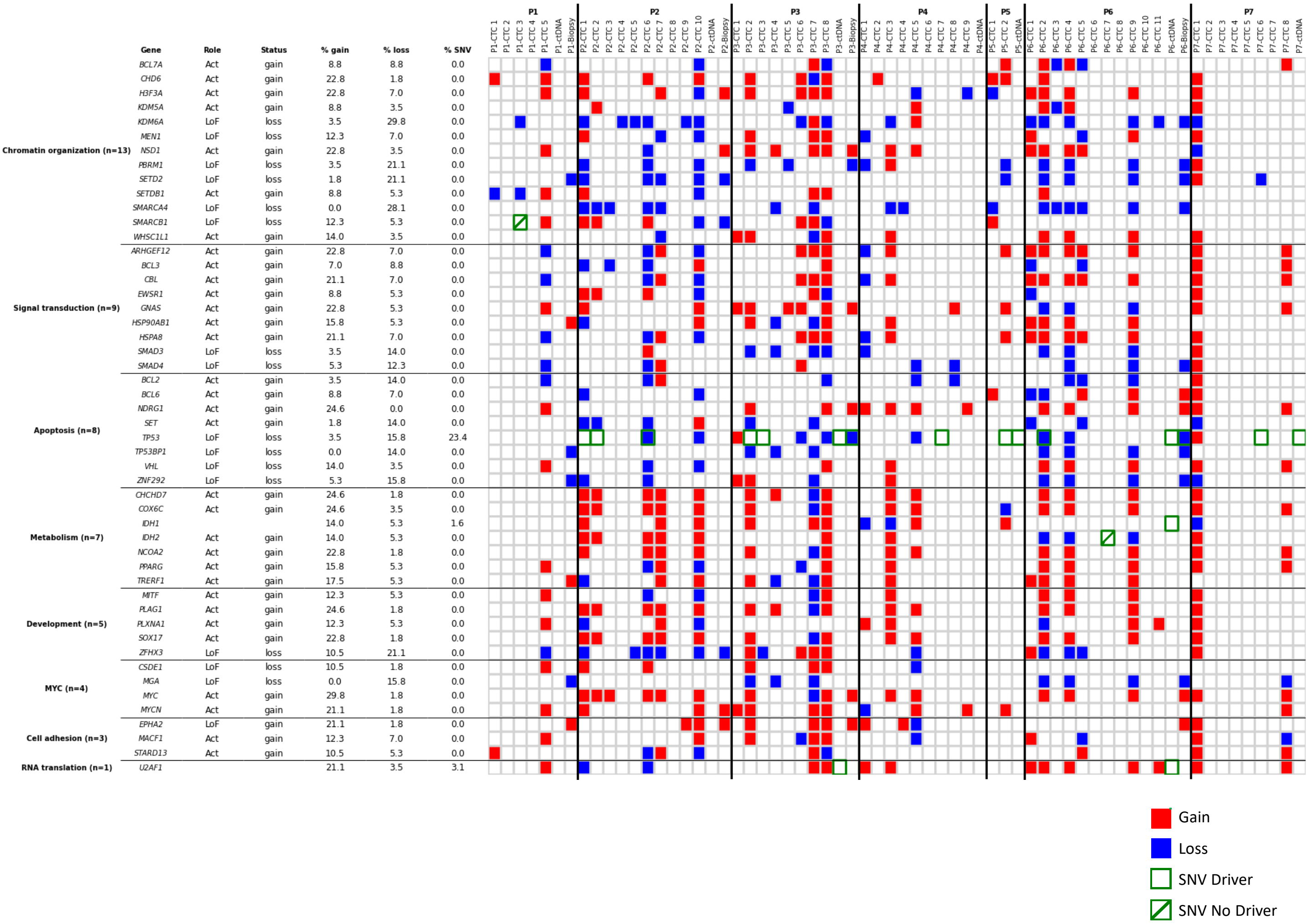

# Supplementary Figure 6

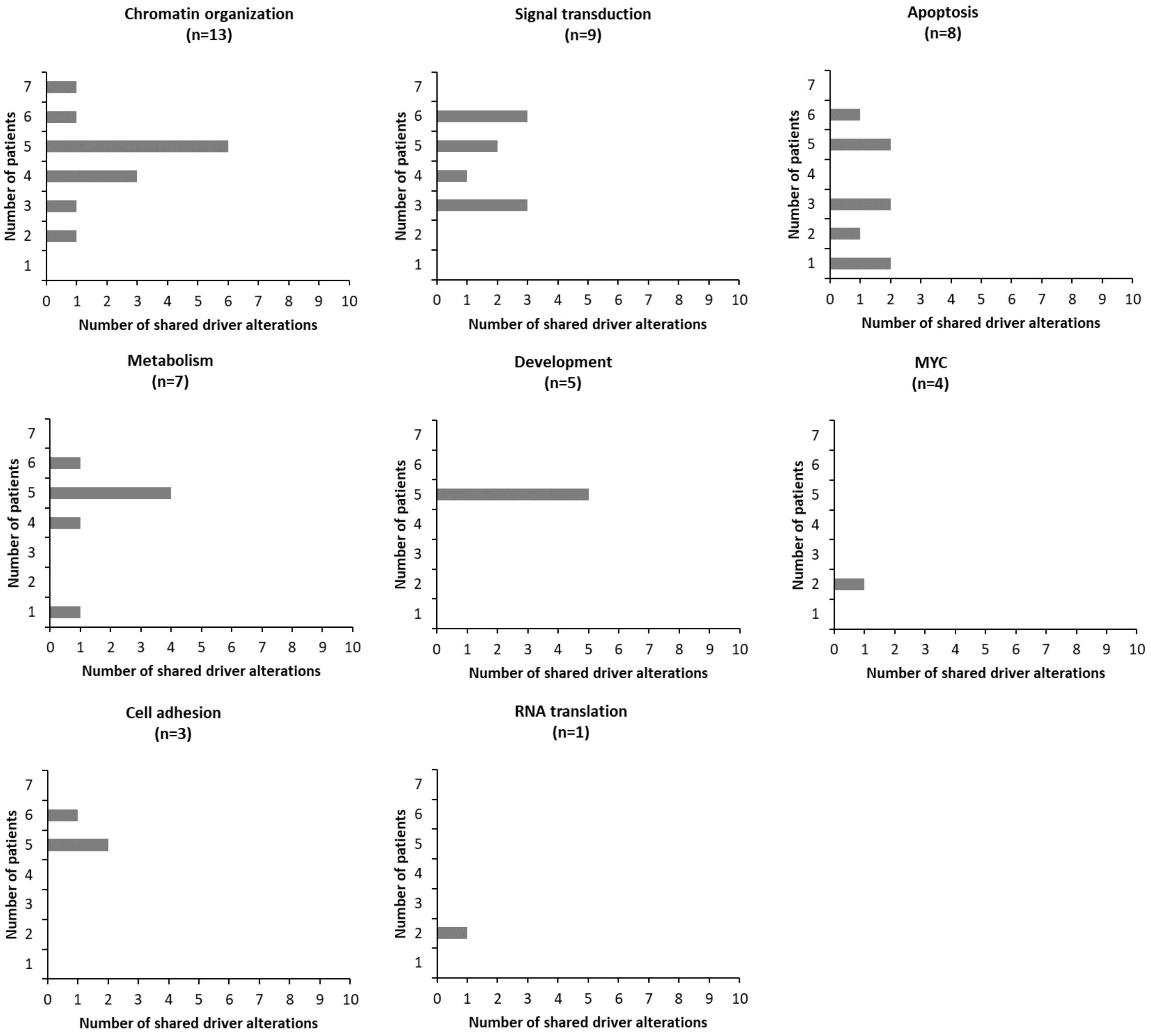

Supplement: Supplementary file 2 — Supplementary Information Figure file [file 41416_2023_2535_MOESM2_ESM.pdf]
