# Supplementary material for: Resistance to BRAF inhibition explored through single circulating tumour cell molecular profiling in BRAF-mutant non-small-cell lung cancer
Source: Br J Cancer. 2024 Jan 4;130(4):682–93. doi: 10.1038/s41416-023-02535-0 (PMC10876548; doi:10.1038/s41416-023-02535-0)
Supplement: Supplementary file 3 — Supplementary Table 1 [file 41416_2023_2535_MOESM3_ESM.docx]

**Supplementary Table 1**. Validation of targeted NGS workflow on cell line harboring *BRAF*^V600E^ mutation

*: Ampli1 Cancer Hotspot Panel Custom Beta contains a total of 315 amplicons including 151 amplicons targeting exons.

**: Coverage Uniformity is the percentage of bases from amplicons covered at >20% of the mean coverage.
