# Supplementary material for: Resistance to BRAF inhibition explored through single circulating tumour cell molecular profiling in BRAF-mutant non-small-cell lung cancer
Source: Br J Cancer. 2024 Jan 4;130(4):682–93. doi: 10.1038/s41416-023-02535-0 (PMC10876548; doi:10.1038/s41416-023-02535-0)
Supplement: Supplementary file 4 — Supplementary Table 2 [file 41416_2023_2535_MOESM4_ESM.docx]

**Supplementary Table 2**. Single CTC WGA, Targeted sequencing and LowPass whole Genome sequencing quality controls

| **Patient** | **CTC Sample** | **WGA GII (/4)** |  | **Targeted sequencing** | | | |  | **LowPass Whole Genome sequencing** | | | |
| --- | --- | --- | --- | --- | --- | --- | --- | --- | --- | --- | --- | --- |
|  |  |  |  | **Mean Depth (X)** | **Median Depth (X)** | **Coverage Uniformity (%)** | **Amplicons**  **with a Depth ≥50X (%)** |  | **Read counts** | **DLRS** | **LMAD** | **R50** |
| P1 | P1-CTC 1 | 2 |  | 1323 | 3 | 27 | 25 |  | 3.593.044 | 0,2622 | 0,37 | 36 |
| P1 | P1-CTC 2 | 2 |  | 1164 | 0 | 40 | 42 |  | 1.589.761 | 0,2361 | 0,22 | 45 |
| P1 | P1-CTC 3 | 2 |  | 1031 | 5 | 43 | 45 |  | 2.743.059 | 0,2206 | 0,20 | 45 |
| P1 | P1-CTC 4 | 4 |  | 1332 | 723 | 62 | 71 |  | 1.802.580 | 0,2137 | 0,20 | 46 |
| P1 | P1-CTC 5 | 1 |  | - | - | - | - |  | 7.178.453 | 0,3685 | 0,33 | 26 |
| P2 | P2-CTC 1 | 4 |  | 1716 | 649 | 59 | 75 |  | 1.204.663 | 0,3865 | 0,94 | 46 |
| P2 | P2-CTC 2 | 3 |  | 3124 | 1903 | 78 | 87 |  | 2.550.046 | 0,2867 | 0,28 | 46 |
| P2 | P2-CTC 3 | 4 |  | 2442 | 1492 | 61 | 80 |  | 1.079.951 | 0,2548 | 0,24 | 46 |
| P2 | P2-CTC 4 | 4 |  | 4436 | 2567 | 56 | 80 |  | 761.513 | 0,2317 | 0,21 | 45 |
| P2 | P2-CTC 5 | 4 |  | 1158 | 316 | 47 | 77 |  | 1.038.093 | 0,3301 | 0,29 | 46 |
| P2 | P2-CTC 6 | 4 |  | 1729 | 876 | 58 | 81 |  | 1.570.291 | 0,2652 | 0,94 | 46 |
| P2 | P2-CTC 7 | 4 |  | 1617 | 559 | 56 | 71 |  | 2.361.962 | 0,3751 | 1,14 | 46 |
| P2 | P2-CTC 8 | 4 |  | 2779 | 1451 | 67 | 79 |  | 1.793.987 | 0,2379 | 0,22 | 46 |
| P2 | P2-CTC 9 | 2 |  | - | - | - | - |  | 2.470.652 | 0,2899 | 0,24 | 44 |
| P2 | P2-CTC 10 | 1 |  | - | - | - | - |  | 224.265 | 0,5249 | 1,12 | 37 |
| P3 | P3-CTC 1 | 4 |  | 1942 | 1053 | 67 | 79 |  | 1.820.220 | 0,3169 | 0,32 | 46 |
| P3 | P3-CTC 2 | 4 |  | 2038 | 1318 | 68 | 85 |  | 2.497.714 | 0,2425 | 0,25 | 46 |
| P3 | P3-CTC 3 | 4 |  | 3317 | 1334 | 47 | 79 |  | 769.621 | 0,2346 | 0,20 | 44 |
| P3 | P3-CTC 4 | 4 |  | 2642 | 1807 | 57 | 87 |  | 772.720 | 0,3011 | 0,27 | 46 |
| P3 | P3-CTC 5 | 4 |  | 2295 | 1040 | 45 | 83 |  | 1.856.764 | 0,2452 | 0,23 | 46 |
| P3 | P3-CTC 6 | 1 |  | - | - | - | - |  | 3.296.945 | 0,3226 | 0,46 | 42 |
| P3 | P3-CTC 7 | 1 |  | - | - | - | - |  | 8.029.104 | 0,3415 | 1,06 | 43 |
| P3 | P3-CTC 8 | 2 |  | - | - | - | - |  | 7.726.693 | 0,2361 | 0,66 | 45 |
| P4 | P4-CTC 1 | 4 |  | 2269 | 1072 | 50 | 83 |  | 1.868.002 | 0,2288 | 0,41 | 45 |
| P4 | P4-CTC 2 | 3 |  | 2234 | 5 | 43 | 44 |  | 757.615 | 0,5261 | 0,45 | 41 |
| P4 | P4-CTC 3 | 4 |  | 1543 | 590 | 55 | 69 |  | 976.034 | 0,2501 | 0,35 | 45 |
| P4 | P4-CTC 4 | 3 |  | 1882 | 13 | 33 | 48 |  | 1.042.124 | 0,2296 | 0,21 | 43 |
| P4 | P4-CTC 5 | 4 |  | 1578 | 347 | 54 | 59 |  | 1.757.724 | 0,2325 | 0,29 | 46 |
| P4 | P4-CTC 6 | 3 |  | 1991 | 219 | 44 | 52 |  | 729.576 | 0,2148 | 0,20 | 45 |
| P4 | P4-CTC 7 | 3 |  | 1725 | 296 | 40 | 57 |  | 2.250.229 | 0,2210 | 0,21 | 45 |
| P4 | P4-CTC 8 | 3 |  | 2326 | 1 | 37 | 42 |  | 2.306.370 | 0,2505 | 0,22 | 44 |
| P4 | P4-CTC 9 | 2 |  | - | - | - | - |  | 1.968.625 | 0,2237 | 0,22 | 43 |
| P5 | P5-CTC 1 | 2 |  | 1264 | 3 | 25 | 27 |  | 1.279.566 | 0,2979 | 0,29 | 41 |
| P5 | P5-CTC 2 | 4 |  | 1666 | 814 | 49 | 71 |  | 1.425.560 | 0,3426 | 0,34 | 46 |
| P6 | P6-CTC 1 | 2 |  | 868 | 0 | 20 | 17 |  | 2.680.837 | 0,3111 | 0,43 | 38 |
| P6 | P6-CTC 2 | 2 |  | 1317 | 5 | 40 | 44 |  | 1.980.659 | 0,2589 | 0,37 | 42 |
| P6 | P6-CTC 3 | 3 |  | 1221 | 0 | 26 | 27 |  | 1.918.563 | 0,2332 | 0,23 | 42 |
| P6 | P6-CTC 4 | 2 |  | 1375 | 0 | 8 | 9 |  | 607.351 | 0,3128 | 0,42 | 27 |
| P6 | P6-CTC 5 | 3 |  | 1560 | 791 | 62 | 70 |  | 1.959.885 | 0,3743 | 0,39 | 46 |
| P6 | P6-CTC 6 | 2 |  | 1440 | 1 | 34 | 34 |  | 1.685.555 | 0,2402 | 0,23 | 42 |
| P6 | P6-CTC 7 | 3 |  | 1280 | 383 | 55 | 62 |  | 1.424.808 | 0,2608 | 0,24 | 46 |
| P6 | P6-CTC 8 | 2 |  | 827 | 0 | 33 | 34 |  | 1.825.041 | 0,3020 | 0,28 | 40 |
| P6 | P6-CTC 9 | 1 |  | - | - | - | - |  | 1.536.271 | 0,2542 | 0,35 | 41 |
| P6 | P6-CTC 10 | 1 |  | - | - | - | - |  | 1.539.918 | 0,2651 | 0,24 | 40 |
| P6 | P6-CTC 11 | 1 |  | - | - | - | - |  | 3.482.215 | 0,2611 | 0,22 | 32 |
| P7 | P7-CTC 1 | 4 |  | 1473 | 321 | 58 | 73 |  | 4.101.450 | 0,2711 | 0,60 | 45 |
| P7 | P7-CTC 2 | 2 |  | 1780 | 1 | 26 | 31 |  | 737.560 | 0,2858 | 0,27 | 40 |
| P7 | P7-CTC 3 | 3 |  | 1268 | 362 | 56 | 62 |  | 1.120.593 | 0,2469 | 0,23 | 46 |
| P7 | P7-CTC 4 | 2 |  | 1590 | 81 | 35 | 50 |  | 764.555 | 0,2297 | 0,21 | 41 |
| P7 | P7-CTC 5 | 3 |  | 1802 | 157 | 48 | 55 |  | 4.996.004 | 0,2283 | 0,21 | 45 |
| P7 | P7-CTC 6 | 3 |  | 1809 | 631 | 56 | 67 |  | 6.056.794 | 0,2288 | 0,21 | 46 |
| P7 | P7-CTC 7 | 4 |  | 2377 | 849 | 57 | 68 |  | 3.997.069 | 0,2318 | 0,21 | 45 |
| P7 | P7-CTC 8 | 1 |  | - | - | - | - |  | 5.740.148 | 0,2792 | 0,29 | 43 |
|  |  |  |  |  |  |  |  |  |  |  |  |  |

Abbreviations: DLRS (*), derivative log ratio spread is a measure of point-to-point consistency or noisiness in copy number along the genome (expected = 0,2-0,3); LMAD (*), absolute deviation from local median copy number (expected = 0,2-0,3); R50, percentage of target (total bp of genome covered) covered by 50% of reads. Low values (<20%) indicate low WGA/library complexity.

* DLRS and LMAD are calculated with a subsampled number of 200.000 reads.
