# Supplementary material for: Resistance to BRAF inhibition explored through single circulating tumour cell molecular profiling in BRAF-mutant non-small-cell lung cancer
Source: Br J Cancer. 2024 Jan 4;130(4):682–93. doi: 10.1038/s41416-023-02535-0 (PMC10876548; doi:10.1038/s41416-023-02535-0)
Supplement: Supplementary file 5 — Supplementary Table 4 [file 41416_2023_2535_MOESM5_ESM.docx]

**Supplementary Table 4**. CNA drivers shared by CTCs and matched tumor biopsy from patients P2, P3 and P6

| **P2  (n=41)** | |  | **P3 (n=36)** | |  | **P6 (n=39)** | |
| --- | --- | --- | --- | --- | --- | --- | --- |
| **Gain (n=31)** | **Loss (n=10)** |  | **Gain (n=32)** | **Loss (n=4)** |  | **Gain (n=14)** | **Loss (n=25)** |
| ACTG1 | CDKN2B |  | ACTG1 | BAP1 |  | BCL6 | ATRX |
| AKAP9 | CHEK2 |  | AHR | BRCA1 |  | CCND2 | BAP1 |
| ALK | NF2 |  | AKAP9 | PBRM1 |  | DLG1 | DCC |
| ARHGEF2 | PRDM1 |  | ASPSCR1 | TP53 |  | EIF4A2 | DLC1 |
| ASH1L | RB1 |  | BCL11B |  |  | EIF4G1 | GPC3 |
| ASPSCR1 | SETD2 |  | BIRC3 |  |  | ETV5 | KDM6A |
| BCL11A | SMARCB1 |  | BRAF |  |  | LPP | MAP2K4 |
| BRAF | TNFAIP3 |  | CANT1 |  |  | MYC | MGA |
| CANT1 | ZFHX3 |  | CAT |  |  | NDRG1 | MLH1 |
| CDK6 | ZNRF3 |  | CCND1 |  |  | PIK3CA | MSR1 |
| CREB3L2 |  |  | CDK6 |  |  | RFC4 | PAX5 |
| EPHA2 |  |  | CREB3L1 |  |  | SOX2 | PBRM1 |
| H3F3A |  |  | CREB3L2 |  |  | SS18L1 | PRDM1 |
| KIAA1549 |  |  | CTTN |  |  | TERT | SETD2 |
| LIFR |  |  | EGFR |  |  |  | SMAD4 |
| MDM4 |  |  | EPHA2 |  |  |  | SMARCA4 |
| MET |  |  | FGFR4 |  |  |  | STK11 |
| MLLT11 |  |  | GNAS |  |  |  | SUFU |
| MNX1 |  |  | HNRNPA2B1 |  |  |  | TGFBR2 |
| MUC1 |  |  | JUN |  |  |  | TNFAIP3 |
| MYCN |  |  | KIAA1549 |  |  |  | TP53 |
| NTRK1 |  |  | LMO1 |  |  |  | TP53BP1 |
| PIK3C2B |  |  | LMO2 |  |  |  | TSC1 |
| PRCC |  |  | MET |  |  |  | WRN |
| RALGDS |  |  | MNX1 |  |  |  | ZNF292 |
| RHEB |  |  | MYC |  |  |  |  |
| RNF213 |  |  | NDRG1 |  |  |  |  |
| SEPT9 |  |  | NSD1 |  |  |  |  |
| SPRR3 |  |  | RHEB |  |  |  |  |
| TPM3 |  |  | RNF213 |  |  |  |  |
| TRIM24 |  |  | SEPT9 |  |  |  |  |
|  |  |  | TRIM24 |  |  |  |  |
|  |  |  |  |  |  |  |  |
